# Integrative Analysis of Inflammatory Response-Related Gene for Predicting Prognosis and Immunotherapy in Glioma

**DOI:** 10.1007/s12031-023-02142-x

**Published:** 2023-07-25

**Authors:** Zhen Zhao, Baoping Zheng, Jianglin Zheng, Yi Zhang, Cheng Jiang, Chuansheng Nie, Xiaobing Jiang, Dongxiao Yao, Hongyang Zhao

**Affiliations:** 1 Department of Neurosurgery, Union Hospital, Tongji Medical College, Huazhong University of Science and Technology, Wuhan, 430022 China; 2 Department of Neonatology, Third Affiliated Hospital of Zhengzhou University, Zhengzhou, 450052 China

**Keywords:** Inflammatory response, Glioma, Prognostic signature, Tumor immune microenvironment, Immunotherapy

## Abstract

Inflammatory response plays a crucial role in the development and progression of gliomas. Whereas the prognostic esteem of inflammatory response-related genes has never been comprehensively explored in glioma, the RNA-seq information and clinical data of patients with glioma were extracted from TCGA, CGGA, and Rembrandt databases. The differentially expressed genes (DEGs) were picked out between glioma tissue and non-tumor brain tissue (NBT). Then, the least absolute shrinkage and selection operator (LASSO) regression analysis was performed to construct the prognostic signature in the TCGA cohort and verified in other cohorts. Kaplan–Meier survival analyses were conducted to compare the overall survival (OS) between the high and low-risk groups. Univariate and multivariate Cox analyses were subsequently used to confirm the independent prognostic factors of OS, and then, the nomogram was established based them. Furthermore, immune infiltration, immune checkpoints, and immunotherapy were also probed and compared between high and low-risk groups. The four genes were also analyzed by qRT-PCR, immunohistochemistry, and western blot trials between glioma tissue and NBT. The 39 DEGs were identified between glioma tissue and NBT, of which 31 genes are associated to the prognosis of glioma. The 8 optimal inflammatory response-related genes were selected to construct the prognostic inflammatory response-related signature (IRRS) through the LASSO regression. The effectiveness of the IRRS was verified in the TCGA, CGGA, and Rembrandt cohorts. Meanwhile, a nomogram with better accuracy was established to predict OS based on the independent prognostic factors. The IRRS was highly correlated with clinicopathological features, immune infiltration, and genomic alterations in glioma patients. In addition, four selective genes also verified the difference between glioma tissue and NBT. A novel prognostic signature was associated with the prognosis, immune infiltration, and immunotherapy effect in patients with gliomas. Thus, this study could provide a perspective for glioma prognosis and treatment.

**Supplementary Information:**

The online version contains supplementary material available at 10.1007/s12031-023-02142-x.

## Introduction

Glioma is the most common type of malignant tumor in the central nervous system, accounting for 40% to 50% of brain tumors, with an estimated annual incidence of 3 to 8 per 100,000 individuals (Gusyatiner and Hegi 2018; Ostrom et al. 2020). Despite progress in comprehensive treatments, such as maximal tumor resection, adjuvant chemotherapy, adjuvant radiotherapy, and immunotherapy, the median survival time of glioma patients is still very short due to the aggressiveness of the glioma, high recurrence rate, and resistance to treatment (Cheng et al. 2020; Guan et al. 2018). Glioma is classified into 4 grades by the World Health Organization (WHO) according to their prognosis and morphological characteristics (Chen et al. 2017). In particular, glioblastoma, with the median overall survival (OS) of only 8 months and the 5-year survival rate of 7.2%, was known as the most malignant glioma (Ostrom et al. 2020; Zheng et al. 2021). Traditionally, the WHO classification is considered an important criterion for the prognosis of gliomas. However, the great differences for the clinical prognosis and treatment effectiveness of patients with the same WHO grade have been found due to the heterogeneity of gliomas (Santangelo et al. 2021). Therefore, to solve this limitation, the construction of new biomarkers for accurately predicting treatment response and prognosis carries great clinical importance for patients with gliomas.

The appearance of white blood cells in tumors was initially discovered by Rudolf Virchow in the nineteenth century, which provides a connection between inflammatory response and cancers. With the rapid development of molecular biology, the important molecular mechanisms of inflammatory response in tumor formation have gradually been elucidated by some studies in recent year (Ikwegbue et al. 2019; Mantovani et al. 2008). Wu et al. indicated that PTPN2 could be induced by inflammatory response and oxidative stress and its deficiency depressed glioma cell growth (Wu et al. 2019). Meanwhile, inflammatory markers, derived from the routinely available parameters in the blood and their derivatives, appear to have the relationship of prognostic value in many tumors including glioma, craniopharyngioma, ad pituitary tumor (Chen et al. 2018a, b; Marinari et al. 2020; Zhao et al. 2021). For instance, some studies reported that the white blood cell had the close relation to the proliferation, migration, immune escape, and prognosis of tumors, which particularly existed in many malignant tumors (McMillan 2009). Likewise, several peripheral blood-derived biomarkers such as the neutrophil/lymphocyte ratio, platelet/lymphocyte ratio, and monocyte-to-lymphocyte ratio have been validated as prognostic inflammatory markers in various types of cancers (Bao et al. 2018; Yang et al. 2016, 2019). Except for the inflammatory response factors in the blood, some inflammatory response-related genes were also used as biomarkers to predict the metastatic possibility and prognosis of some cancers (Budhu et al. 2006; Lin et al. 2021). However, interestingly, the relationship between inflammatory response-related genes and the prognosis of patients with gliomas remains unclear.

Immunotherapy, which emerged in recent years, is considered to be one of the most promising treatment methods. For example, a variety of therapeutic antibodies blocked the immune checkpoints, such as programmed cell death protein 1 (PD1) and cytotoxic T lymphocyte-associated antigen 4 (CTLA4), which have demonstrated superior performance in the treatment of many tumors such as glioma, liver cancer, and non-small-cell lung cancer (Johnston and Khakoo 2019; Xu et al. 2020). Unfortunately, immunotherapy based on immune checkpoints is not always useful for patients due to the heterogeneity of tumors and the complex tumor microenvironment. Therefore, it is greatly crucial and urgent to find more immunotherapy targets and clarify the molecular mechanism of more detailed immunotherapy response.

With the tremendous progress of modern sequencing technology, many RNA-seq transcriptome data of cancers can be found in some public databases, such as TCGA, CGGA, and Rembrandt databases. In this study, we systematically analyzed RNA-seq data and clinical data of glioma patients from these databases. Based on 8 inflammatory response-related genes, a prognostic inflammatory response-related gene signature (IRRS) of glioma patients was constructed in the TCGA database. Meanwhile, a reliable predictive nomogram model incorporating IRRS and clinicopathological characteristics was established and validated in the independent database. In addition, the correlations of IRRS with immune landscape, tumor mutation burden, and the efficacy of immunotherapy were investigated. We consider that the IRRS has potential in patient management and can serve as potential therapeutic biomarkers for glioma in the clinical practice.

## Materials and Methods

### Patient Data Collection

The RNA-seq transcriptome data and corresponding clinical information of patients with glioma were extracted from TCGA, CGGA, and Rembrandt databases. The RNA-seq data of TCGA and CGGA were quantified with fragments per kilobase of transcript per million mapped reads standardized by using the RSEM algorithm, whereas the Rembrandt dataset was normalized microarray format (Zheng et al. 2021). Patients without survival data or OS less than one month were excluded from analysis for which they might die of the acute complications, rather than of the glioma itself. Therefore, a total of 1896 glioma patients were included in this study. The TCGA dataset (*n* = 631) was used as the training cohort; the CGGA-693 dataset (*n* = 656), CGGA-325 dataset (*n* = 309), and Rembrandt dataset (*n* = 300) were used as the verification cohorts. The clinicopathological characteristics of the included glioma patients are shown in Table 1. Then, the RNA-seq transcriptome information of non-tumor brain tissue (NBT) samples was searched from the GTEx database. In addition, the 200 inflammatory response-related genes were collected from the Molecular Signatures Database V7.4 (https://www.gsea-msigdb.org/gsea/msigdb). Further details on these genes are summarized in Supplementary Table S1. If the gene expression data were discovered in less than half of the samples, the gene was excluded in order to facilitate the subsequent data analysis.

**Table 1:** Characteristics of glioma patients in training and validation cohorts

Clinicopathological characteristics	Training cohort	Validation cohorts		
	TCGA (631)	CGGA-693 (656)	CGGA-325 (309)	Rembrandt (300)
Age (years)				
< 45	296	357	178	110
> = 45	336	298	131	181
NA	0	1	0	9
Gender				
Female	243	282	115	92
Male	333	374	194	146
NA	55	0	0	62
WHO grade				
II	201	172	97	66
III	222	248	73	57
IV	153	236	135	144
NA	65	0	4	33
Histology				
Astrocytoma	154	NA	NA	NA
Oligoastrocytoma	110	NA	NA	NA
Oligodendroglioma	159	NA	NA	NA
Glioblastoma	153	236	135	NA
NA	55			
IDH status				
Mutant	401	332	165	NA
Wild type	221	276	143	NA
NA	9	48	1	NA
1p19q codeletion				
Codel	157	137	62	12
Non-codel	468	453	239	91
NA	6	66	8	197
MGMT promoter status				
Methylated	449	304	151	NA
Unmethylated	150	217	140	NA
NA	32	135	18	NA

### Identification of Differentially Expressed Genes (DEGs) and Consensus Cluster

The DEGs between TCGA cohort and h GTEx cohort were identified through the differential-expression analysis. The condition that log2 fold-change |logFC|> 1 and an adjusted false-discovery rate FDR < 0.05 were regarded as the cutoff values. Furthermore, Search Tool for the Retrieval of Interacting Genes (STRING) software (http://www.string-db.org/) was used to analyze the interactions and evaluate the level of interactions among the above DEGs.

In order to investigate the best gene subgroups, the glioma patients of TCGA were divided into different clusters based on the expression of DEGs. The resampling procedure was used to sample 80% of the sample for 50 times, the similarity distance between the samples was estimated by Euclidean distance, and the “km dist” as a clustering algorithm was used to select reliable and stable subgroup classification (Ghosh and Barman 2016). The “proportion of ambiguous clustering” was used to select an optimal value of clusters with the criteria that the clusters have high consistency, and the area under the cumulative distribution function curve does not increase significantly (Zheng et al. 2020).

### Development and Validation of IRRS

The univariate Cox regression was conducted to explore the relationship between DEGs and prognosis of patients (*p* < 0.05 was used as the significance threshold). Then, a prognostic model was constructed through the least absolute shrinkage and selection operator (LASSO) regression which prevented to minimize the risk of overfitting (Sauerbrei et al. 2007; Simon et al. 2011), which was performed within the TCGA cohort. In order to explore the value of *λ* corresponding to the lowest partial likelihood deviance, the ten-fold cross-validation was used to select the optimal penalty parameter *λ* of the prognostic model. Subsequently, the risk score of each patient was calculated in the light of the regression coefficient of the model and the expression of the corresponding gene. The calculation formula is shown below:

$$\mathrm{Riskscore}=\sum_{i=1}^n\left(Coef_{\mathrm i}\ast{\mathrm X}_{\mathrm i}\right)$$

where $n$ represents the number of all the selected gene; $i$ represents the serial number of each gene; ${X}_{i}$ and ${\text{Coef}}_{i}$ refer to the expression level of each selected gene and corresponding coefficient, respectively. The median risk score was considered as the cutoff value to divide the patients into high or low-risk group. The Kaplan–Meier survival curve analysis with log-rank test was performed to assess the ability of predicting OS between the high- and low-risk groups. The receiver operating characteristic (ROC) curve and the area under the ROC curve (AUC) were calculated to evaluate the accuracy of the inflammatory response-related gene. The above analyses were performed simultaneously in the training and validation cohorts.

### Establishment and Evaluation of Nomogram

The univariate and multivariate Cox regression analyses were performed for identifying the independent prognostic factors in terms of the risk scores and clinicopathological characteristics. Next, a nomogram was established in the TCGA cohort based on the independent prognostic factors. The calibration curves and C-index were performed in the training and validation cohorts to evaluate the availability of this nomogram (Harrell et al. 1996). In addition, the ROC curves were also plotted to assess the accuracy of the nomogram for OS prediction in the training and validation cohorts.

### Functional Enrichment Analysis

The DEGs between high and low-risk groups were identified with the criteria of |log2FC|> 2 and adjusted *p* < 0.05 by BH method. Then, Gene Ontology (GO) and Kyoto Encyclopedia of Genes and Genomes (KEGG) analyses were conducted to predict the function of these DEGs.

### Evaluation of Tumor Immune Microenvironment

The immune scores and stromal scores, calculated using the ESTIMATE algorithm, were used to analyze the infiltration levels of immune cells and stromal cells in glioma tissues (Yoshihara et al. 2013). CIBERSORT, a deconvolution algorithm with 1000 permutations, was applied to estimate the abundance of 22 immune cells (Chen et al. 2018a, b). Patients with CIBERSORT *p* ≥ 0.05 were excluded from the subsequent analysis. These immune cells included as follows: resting memory CD4 + T cells, activated memory CD4 + T cells, Tfh cells, Tregs, γδ T cells, CD8 + T cells, naive CD4 + T cells, naive B cells, memory B cells, plasma cells, resting NK cells, activated NK cells, macrophages (M0, M1, and M2), resting DCs, activated DCs, resting mast cells, activated mast cells, eosinophils, neutrophils, and monocytes. We evaluated the differences of the abundance of 22 immune cells and the expression levels of immune-related molecules between the high and low-risk groups.

### Evaluation of Genomic Alterations and Immunotherapy

Tumor mutation burden (TMB) has been defined as the total number of somatic, coding, base substitution, and indel mutations per megabase of genome examined. In this study, we selected a novel TME-scoring algorithm to explore the differences in TME between the high- and low-risk groups. Furthermore, the tumor immune dysfunction and exclusion (TIDE) algorithm was applied to evaluate the predictive efficiency of risk scores for the immune checkpoint inhibition (ICI) response in glioma (Jiang et al. 2018). In addition, the distributions of risk scores between non-respond and respond groups and the differences of response rates between high- and low-risk groups are also analyzed and compared.

### Quantitative Real-Time Polymerase Chain Reaction (qRT-PCR), Immunohistochemistry (IHC), and Western Blot

Normal human astrocyte line HA1800 and human glioma cell lines U87, U251, A172, LN229, KNS89, and T98G were purchased from the Cell Bank of the Chinese Academy of Sciences. All cells were cultured in Dulbecco’s modified Eagle’s medium (Corning, USA) supplemented with 10% fetal bovine serum (Gibco, USA) and 1% penicillin/streptomycin in a humidified incubator with 5% carbon dioxide at 37 °C. Clinical specimens of 10 glioma patients were collected in the Department of Neurosurgery, Wuhan Union Hospital, from May 2020 to October 2021. During the same period, 10 samples from acute brain injury patients were taken as the control group. Glioma tissue and NBT from the patients were immediately frozen in liquid nitrogen and then stored at −80 °C before further analysis. The study was approved by the ethics committee of Wuhan Union Hospital, and written informed consent was obtained from each patient.

The total RNA was extracted from sample tissues and cell lines using RNAiso Plus (Takara 9109). cDNA was synthesized by reverse transcription through using HiScript^®^ III RT SuperMix for qPCR (+ gDNA wiper) (Vazyme R323-01) according to the instruction. The qRT-PCR analyses were performed using the AceQ^®^ qPCR SYBR Green Master Mix (Vazyme Q111-02) and PCR LightCycler480 (Roche Diagnostics, Basel, Switzerland). All expression data was normalized to GAPDH as an internal control using the 2–ΔΔCt method. Then, we verified the protein levels of the selected genes by IHC experiments. Paraffin-embedded clinical tissue specimens were sectioned, dewaxed, dehydrated, and washed with 3% hydrogen peroxide in methanol. 3% bovine serum albumin was incubated in phosphate buffer for 30 min to block non-specific binding. Subsequently, sections were incubated overnight at 4 °C with primary antibodies against GABBR1, CALCRL, SERPINE1, and MMP14. These sections were then accepted to three mild washes in PBS for 10 min and stained with secondary antibody for 60 min. After the addition of diaminobenzidine, the sections were counterstained with hematoxylin and turned blue in 1% ammonia. Finally, the samples were sealed, observed, and photographed by light microscopy. The intensity of positive staining for GABBR1, CALCRL, SERPINE1, and MMP14 in glioma and NBT sections was detected using Image-Pro Plus 6.0 software. All images were taken with the same microscope and camera. In addition, the selective genes also verified the difference by western blot trials in between glioma and NBT.

### Statistical Analysis

The statistical analyses and graph visualization were conducted by the R software (version 4.0.1, http://www.R-project.org). The PERL programming language (version, 5.30.2, http://www.perl.org) was used to deal with RNA-seq information. The chi-square test or two-sided Fisher’s exact test was performed to compare the categorical variables between the high and low-risk groups; similarly, correlations between risk score and clinicopathological parameters were the same methods. The *t*-test or one-way ANOVA test was utilized to compare the continuous variables with normal distribution between two groups or more than two groups, while Mann–Whitney *U* test was used to compare continuous variables with non-normal distribution between two groups, and Kruskal Wallis test was used to compare continuous variables with non-normal distribution in more than two groups. The cutoff value with statistical significance was set at two-tailed *p* < 0.05.

## Results

### Identification of the DEGs Between Glioma Tissue and NBT

**Fig. 1:**
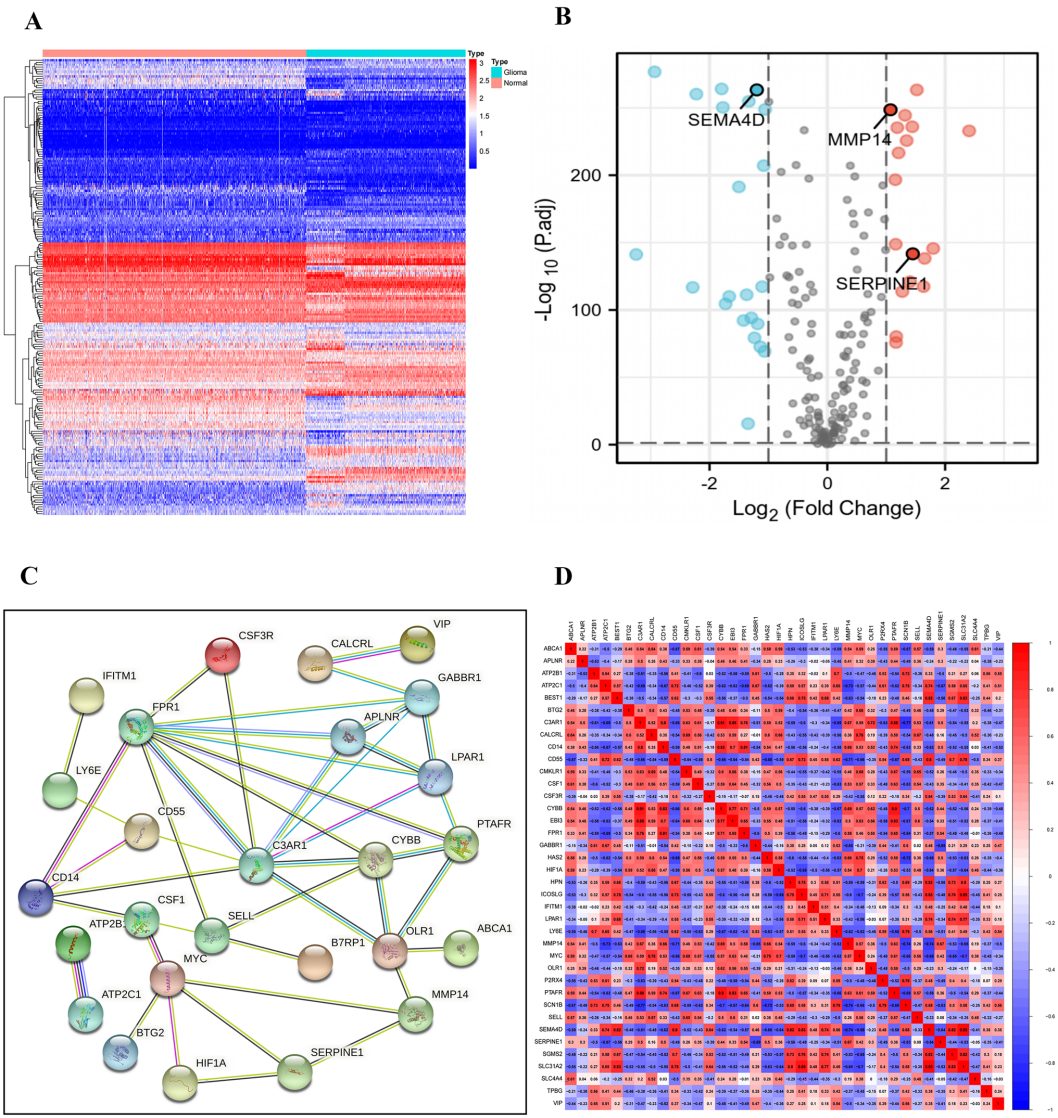
Identification and analysis of DEGs between glioma tissue and NBT. **A** Heatmap and **B** volcano plot showed the DEGs between glioma tissue and NBT. The red, green, and black dots represented the upregulated genes, downregulated genes, and no difference, respectively. **C** Network and **D** correlations among 39 DEGs

These data were preprocessed before identifying the DEGs, the normalization and batch effect removal from GTEx and TCGA cohorts was conducted. 39 DEGs were identified between glioma samples and NBT samples, with the threshold levels of significance including the absolute value of the |logFC|> 1 and FDR < 0.05. Among the identified DEGs, 17 upregulated genes and 22 downregulated genes were identified, which indicated that inflammatory response-related genes exerted important biological functions in the tumorigenesis of glioma. The heatmap and volcano plot of the DEGs are showed in Fig. 1A, B. In addition, the network and correlation analysis of 39 inflammatory response-related genes was performed to identify their interactions (Fig. 1C, D).

### The Clinical Characteristics and Prognosis of Patients with Different Clusters

**Fig. 2:**
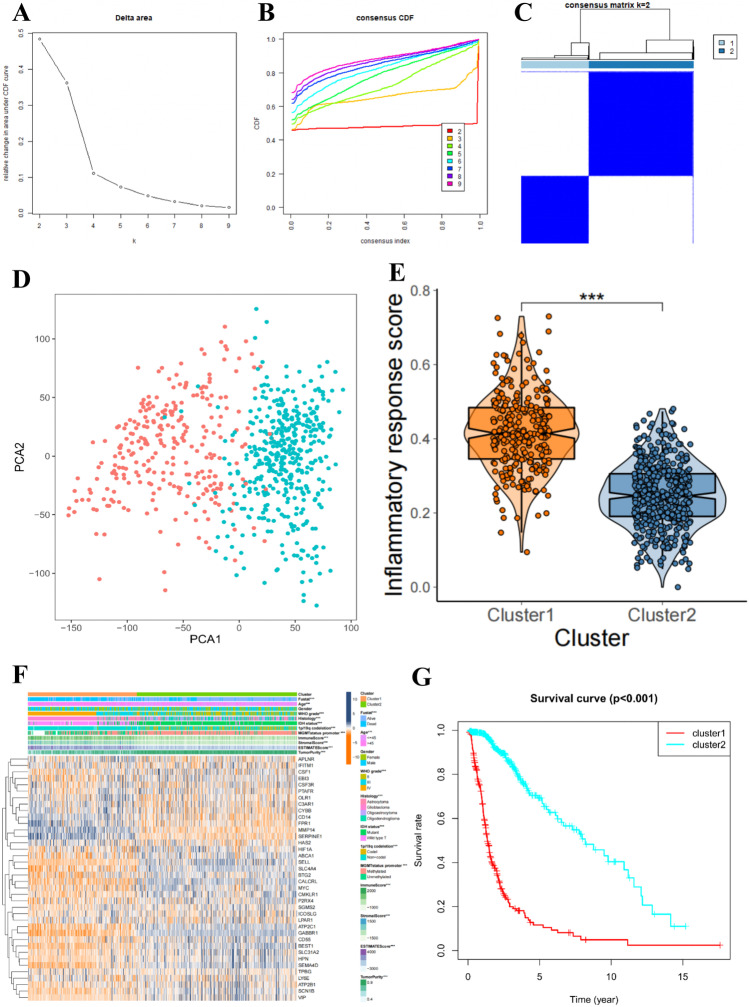
The clinical characteristics and prognosis of patients with different clusters. **A** Relative change in area under CDF curve for *k* = 2 to 10. **B** Consensus clustering cumulative distribution function for *k* = 2 to 10. **C** Consensus clustering matrix for *k* = 2. **D** Principal component analysis between cluster 1 and cluster 2 groups. **E** Inflammatory response scores were significantly higher in cluster 1 patients than in cluster 2 patients. **F** Heatmap and clinicopathologic features of the two clusters defined by the inflammatory response-related gene consensus expression. **G** Kaplan–Meier survival curves showed the significantly shorter OS in the cluster 1 group than the cluster 2 group

In line with the expression similarity of inflammatory response-related genes and the criteria for selecting the number of clusters, *k* = 2 was considered as the most appropriate value with clustering stability increasing from *k* = 2 to 10 in the TCGA datasets (Fig. 2A, B). Hence, the patients from TCGA cohort were divided into two clusters (Fig. 2C). Subsequently, the principal component analysis (PCA) was used to compare the transcriptional profile between cluster 1 and cluster 2 subgroups. The results displayed that there is a clear boundary between the two subgroups (Fig. 2D). Inflammatory response scores were quantified for each patient using the ssGSEA method and were significantly higher in cluster 1 patients than in cluster 2 patients (Fig. 2E). Moreover, the clinicopathological characteristics, survival status, and immune scores between different clusters were identified, and 39 inflammatory response-related gene expression values were screened out as a heatmap (Fig. 2F). The cluster 1 group is significantly correlated with dead (*p* < 0.001), older patients (*p* < 0.001), higher grade (*p* < 0.001), glioblastoma and astrocytoma (*p* < 0.0001), IDH-wild type (*p* < 0.001), 1p19q-non-codel (*p* < 0.001), unmethylated MGMT status promoter (*p* < 0.001), high immune score (*p* < 0.001), high stromal score (*p* < 0.001), high ESTIMATE score (*p* < 0.001), and low tumor purity (*p* < 0.001), while cluster 2 group is significantly correlated with the opposite results. In order to compare the survival distributions of the two clusters, the Kaplan–Meier survival curves showed the significantly shorter OS in the cluster 1 group than the cluster 2 group (Fig. 2G, p < 0.001).

### Development and Verification of IRRS

**Fig. 3:**
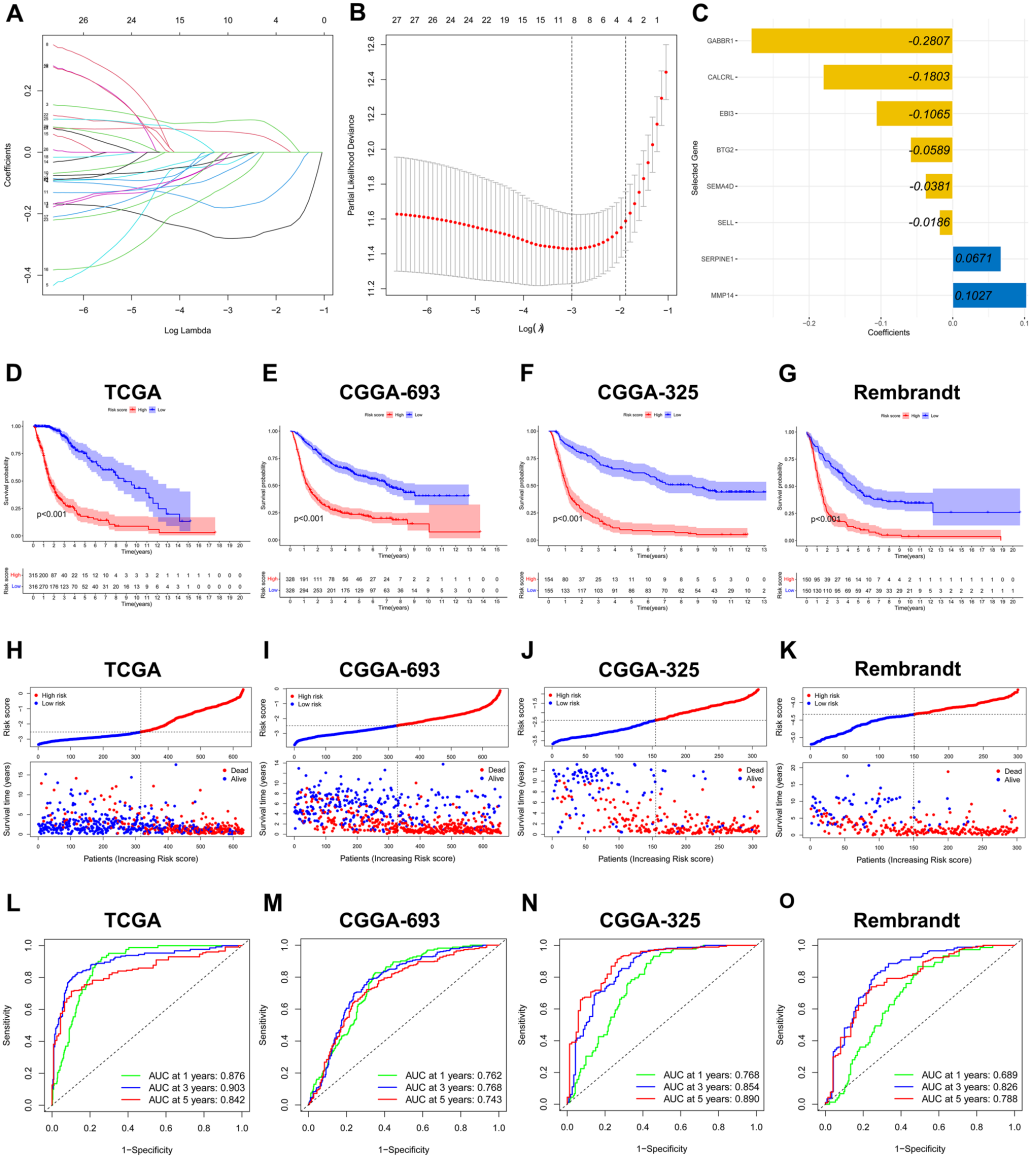
Construction and validation of the IRRS. **A**, **B** The LASSO regression was performed to minimize the risk of overfitting with the minimum criteria. **C** The LASSO coefficients of 8 optimal inflammatory response-related genes. **D**–**G** The Kaplan–Meier curves of the TCGA, CGGA-693, CGGA-325, and Rembrandt cohorts, respectively. **H**–**K** The distribution plots of the risk score and survival status in the TCGA, CGGA-693, CGGA-325, and Rembrandt cohorts, respectively. **L**–**O** The ROC curves of IRRS in predicting 1-, 3-, and 5-year OS in the TCGA, CGGA-693, CGGA-325, and Rembrandt cohorts, respectively

The 31 genes were obviously related to the OS of glioma based on the DEGs, and they replenished in Supplementary Table S2 (*p* ≤ 0.05). Among these 31 genes, 22 were protective factors and 9 were risk factors for prognosis. Then, 8 optimal prognostic genes were acquired and incorporated in the IRRS by the LASSO regression analysis, including GABBR1, CALCRL, EBI3, BTG2, SEMA4D, SELL, SERPINE1, and MMP14 (Fig. 3A, B). There were six genes (GABBR1, CALCRL, EBI3, BTG2, SEMA4D, and SELL) related to good prognosis, while SERPINE1 and MMP14 were the opposite (Fig. 3C and Supplementary Fig. S1). The risk scores of patients were calculated as follows: risk score = (−0.2807 × expression value of GABBR1) + (−0.1803 × expression value of CALCRL) + (−0.1065 × expression value of EBI3) + (0.0589 × expression value of BTG2) + (−0.0381 × expression value of SEMA4D) + (−0.0186 × expression value of SELL) + (0.0671 × expression value of SERPINE1) + (0.1027 × expression value of MMP14). Subsequently, the median risk score, as the cutoff value, was used to stratify the glioma patients into the high- and low-risk groups. In the TCGA cohort, the Kaplan–Meier curve demonstrated that the survival time of patients in the low-risk group was significantly longer than that in the high-risk group (log-rank test *p* < 0.001; Fig. 3D). The distribution plot of the risk score and survival status showed that the risk score had the positive correlation with the deaths of glioma patients (Fig. 3H). A satisfactory predictive performance of the prognostic model was confirmed by the AUC for 1-, 3-, and 5-year OS (AUC = 0.876, 0.903, and 0.842, respectively; Fig. 3L).

To test the stability of the prognostic signature constructed on the TCGA cohort, the same analyses were performed in the CGGA-693 cohort, CGGA-325 cohort, and Rembrandt cohort. Similarly, patients in the low-risk group had longer survival time than those in the high-risk group (Fig. 3E–G and I–K). Furthermore, the AUCs for predicting the 1-, 3-, and 5-year survival were 0.762, 0.768, and 0.743 in the CGGA-693 cohort, respectively (Fig. 3M), the AUCs for predicting the 1-, 3-, and 5-year survival were 0.768, 0.854, and 0.890 in the CGGA-325 cohort, respectively (Fig. 3N), and the AUCs for predicting the 1-, 3-, and 5-year survival were 0.689, 0.826, and 0.788 in the Rembrandt cohort, respectively (Fig. 3O). Based on these results, we confirmed the accuracy and stability of predictive signature.

### The Relationship Between IRRS and Clinicopathological Characteristics

**Fig. 4:**
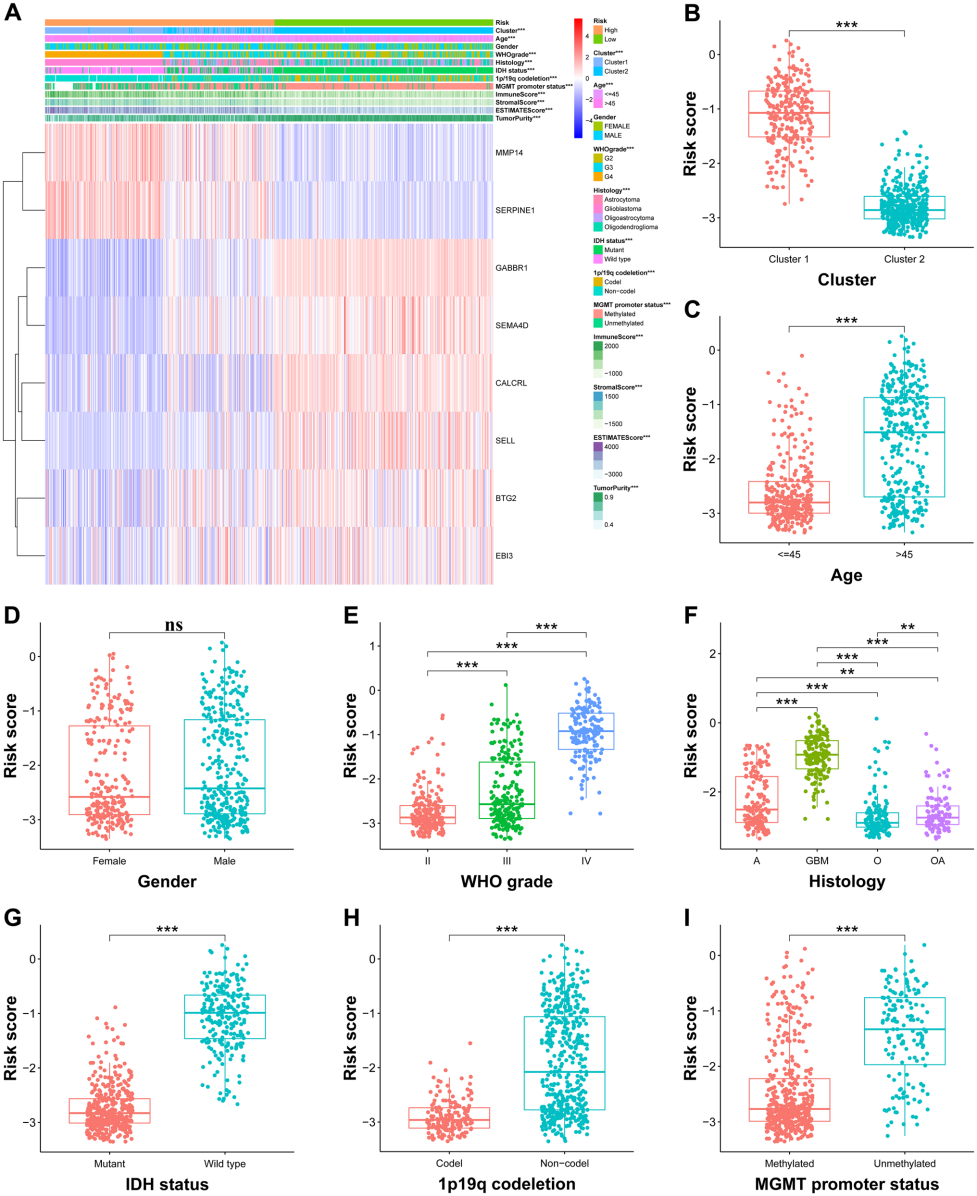
Correlation analysis between IRRS and clinicopathological characteristics in the TCGA cohort. **A** Heatmap represented expression levels of 8 selected inflammatory response-related genes and the distribution of clinicopathological characteristics in the high- and low-risk groups, respectively. **B**–**I** Different levels of risk scores in glioma patients stratified by cluster, age, gender, WHO grade, histology, IDH status, 1p19q codeletion, and MGMT promoter status. A, astrocytoma; O, oligodendroglioma; OA, oligoastrocytoma; GBM, glioblastoma. *p < 0.05, **p < 0.01, ***p < 0.001, and ns no significance

The association of the 8 selected genes and clinicopathologic parameters between high- and low-risk groups was analyzed in the TCGA cohort, and the results showed that the dangerous genes were upregulated in the high-risk group and the protective genes were upregulated in the low-risk group (Fig. 4A). Meanwhile, the differences of age, gender, histology, WHO grade, IDH status, 1p19q codeletion, and MGMT promoter status between high- and low-risk groups were compared in all training cohort and validation cohorts and showed in Supplementary Tables S3–S6. In addition, the values of risk score between cluster 1 and cluster 2 and the clinicopathological characteristics were also compared. In the TCGA cohort, glioma patients of the cluster 1 subgroup with the clinicopathological characteristics, age > 45 years, more malignant type of histology, higher WHO grade, IDH wild type, 1p19q codeletion non-codel, and MGMT promoter unmethylated showed significantly higher levels of risk score, while no differences were observed between patients satisfied by gender (Fig. 4B–I). Likewise, the risk score of glioma patients in the CGGA-693, CGGA-325, and Rembrandt cohorts was identified the similar results to the TCGA cohort (Supplementary Fig. S2).

The subgroup survival analyses were performed to explore whether the clinicopathological characteristics would affect the predictive accuracy of IRRS. The results indicated that patients with risk score was negatively related to the survival outcomes in all subgroups, but for the WHO grade IV subgroup within the TCGA cohort (Supplementary Fig. S3). The other cohorts showed the same results but the IDH-wild subgroup in the CGGA-693 cohort and the 1p19q-non-codel subgroup in the Rembrandt cohort (Supplementary Fig. S4).

### Establishment and Evaluation of Nomogram

**Fig. 5:**
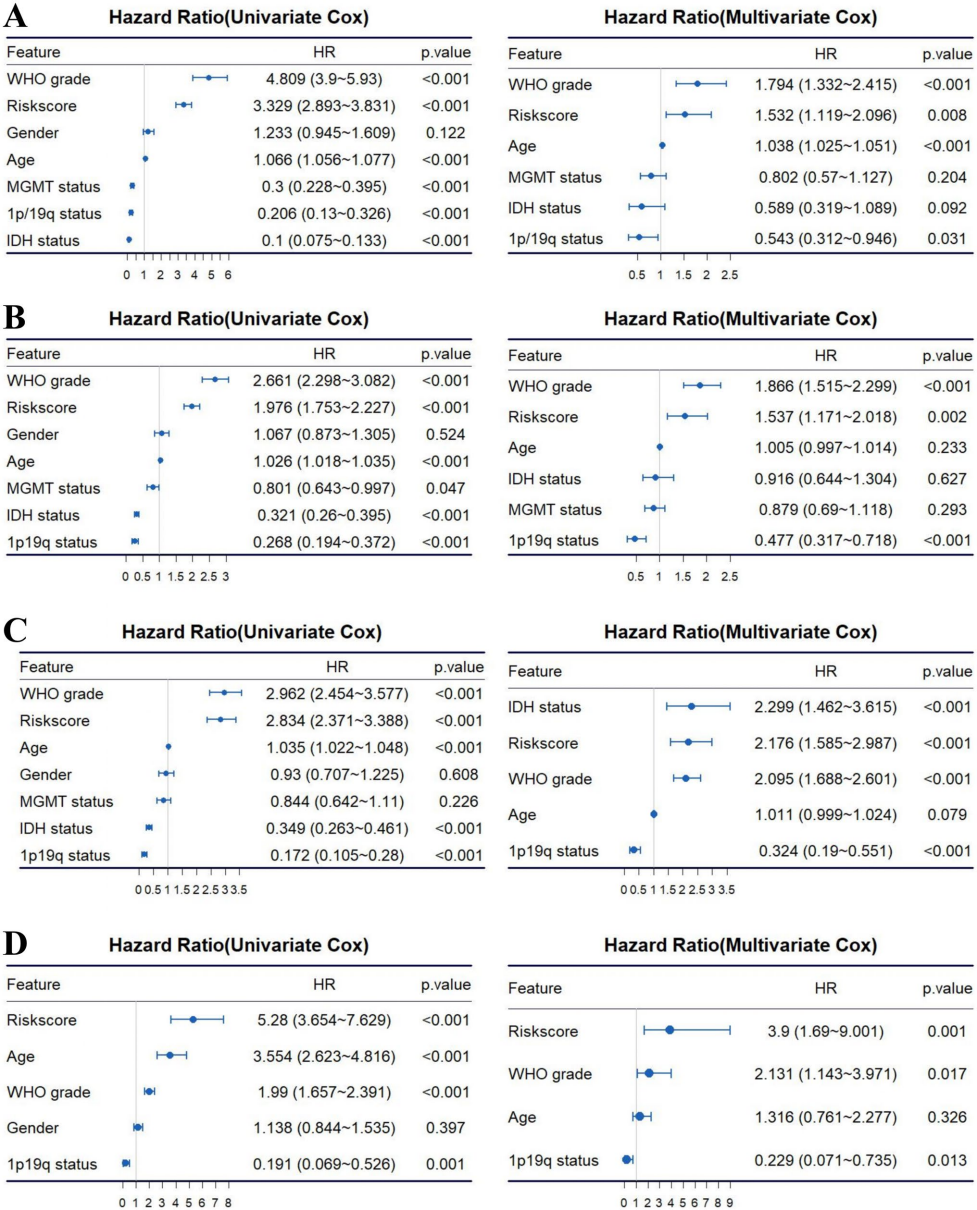
The univariate Cox regression and multivariate Cox regression were performed on four variables including age, gender, WHO grade, IDH status, 1p19q codeletion, and MGMT promoter status. **A**–**D** The univariate Cox regression and multivariate Cox regression for identifying the OS-related factors in the TCGA, CGGA-693, CGGA-325, and Rembrandt cohorts, respectively

**Fig. 6:**
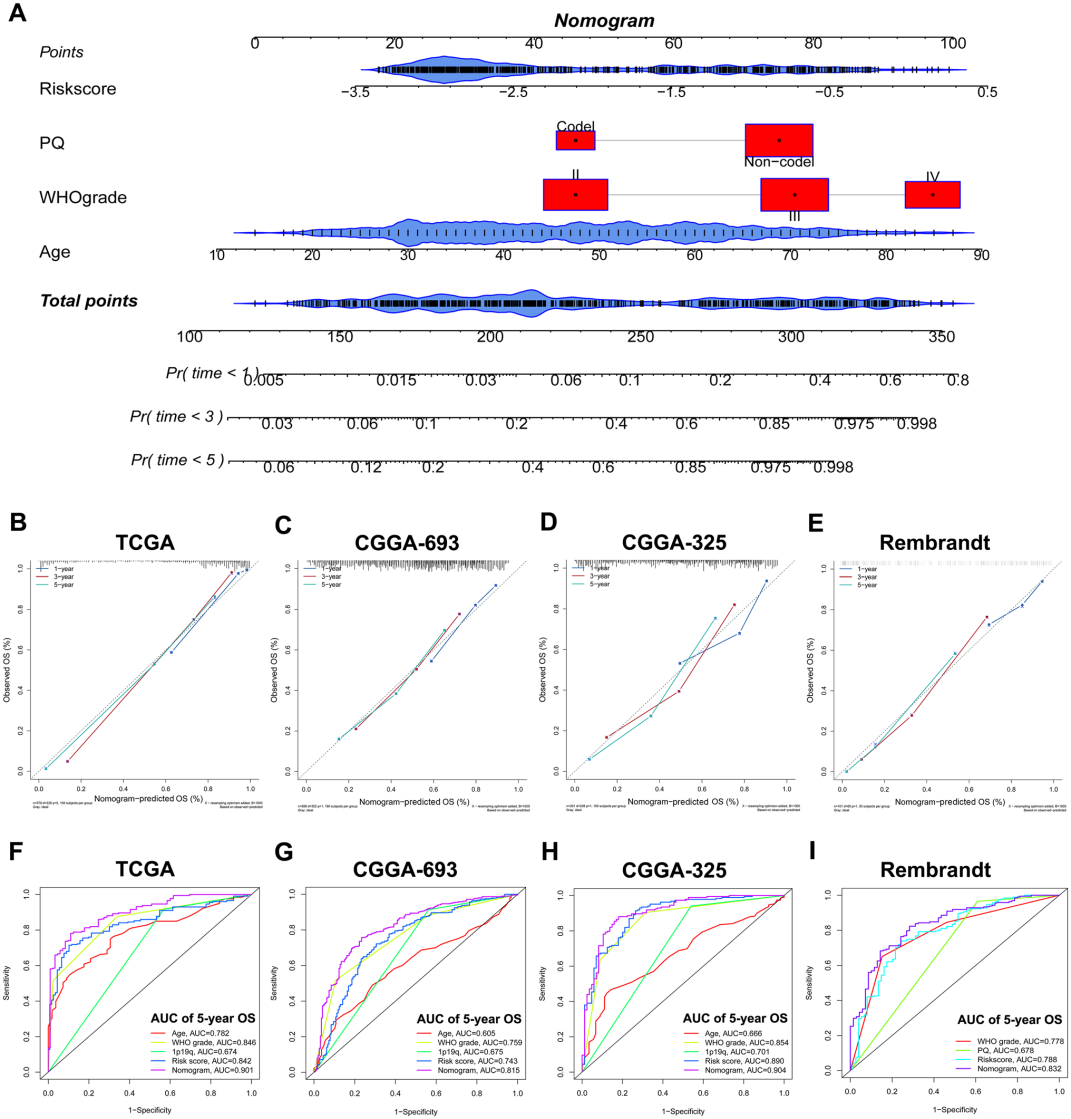
Establishment and evaluation of a nomogram in the TCGA cohort. **A** Nomogram comprised IRRS, age, WHO grade, and 1p19q codeletion. **B**–**E** Calibration curves showed the good consistency between predicted and observed 1-, 3-, and 5-year OS in the TCGA, CGGA-693, CGGA-325, and Rembrandt cohorts, respectively. **F**–**I** The ROC curves of the nomogram predicted 5-year OS in the TCGA, CGGA-693, CGGA-325, and Rembrandt cohorts, respectively

The univariate Cox regression and multivariate Cox regression were analyzed subsequently for identifying the OS-related factors in the TCGA (Fig. 5A), CGGA-693 (Fig. 5B), CGGA-325 (Fig. 5C), and Rembrandt cohorts (Fig. 5D). And the risk score based on 8 selected genes was confirmed as an independent prognostic factor without being affected by other clinicopathological characteristics. Then, the independent prognostic factors in the TCGA cohort (age, WHO grade, 1p19q, and risk score) were used to establish the nomogram for predicting the 1-, 3-, and 5-year prognosis of the glioma patients (Fig. 6A). The internal evaluation was initially performed. The C-index was 0.857 (95% CI: 0.812–0.896), and the calibration curves indicated a great coordinate between the actual and nomogram-predicted probability of 1-, 3-, and 5-year OS (Fig. 6B). The ROC curve presented that the nomogram acquired the highest predictive accuracy of 5-year OS than risk score or clinicopathological features (Fig. 6F). Likewise, the external validation of this nomogram was performed in the CGGA-693, CGGA-325, and Rembrandt cohorts. The C-indices were 0.794 (95% CI: 0.757–0.831), 0.833 (95% CI: 0.781–0.885), and 0.849 (95% CI: 0.730–0.968) in the CGGA-693, CGGA-325, and Rembrandt cohorts, respectively. The calibration curves all presented a satisfactory consistency between the actual and nomogram-predicted probability of 1-, 3-, and 5-year OS in these three cohorts (Fig. 6C–E). Furthermore, the ROC curve illustrated that the nomogram had the highest predictive accuracy of 5-year OS than risk score or clinicopathological features in three validation cohorts (Fig. 6G–I). Meanwhile, the ROC curve analysis also exhibited that the nomograms had the highest accuracy in predicting 1- and 3-year OS in comparison to other independent prognostic factors (Supplementary Fig. S5). Thus, the nomogram has the potential as a quantitative method to predict the prognosis of patients with glioma.

### Functional Enrichment Analysis

**Fig. 7:**
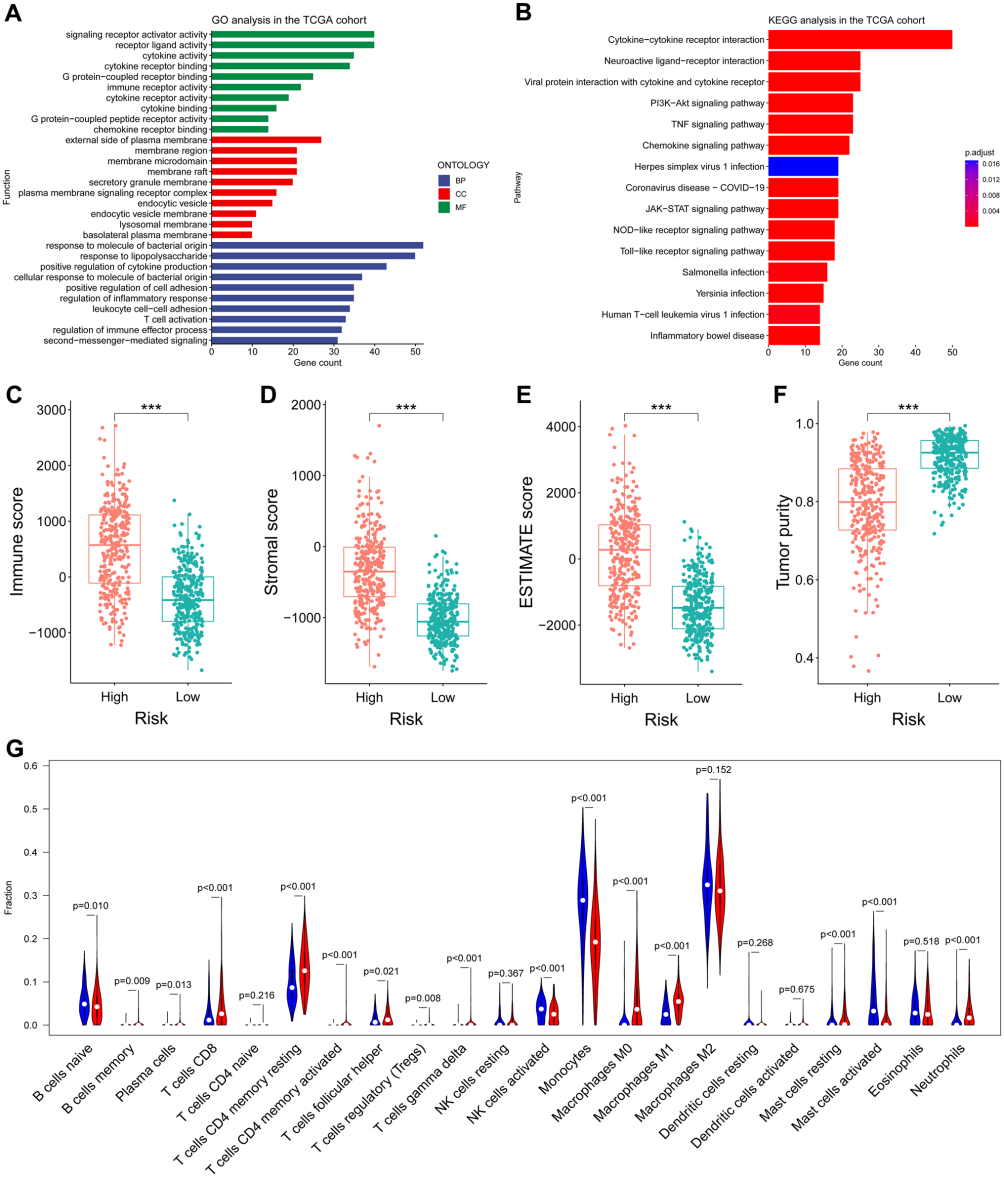
Functional enrichment analysis and glioma immune microenvironment. **A**, **B** GO analysis and KEGG analysis in the TCGA cohort. **C**–**F** Comparison of immune scores, stromal scores, ESTIMATE scores, and tumor purity between the high and low-risk groups. **E** The abundance of 22 immune cells in the high and low-risk groups

The GO function and KEGG pathway enrichment analyses were performed to characterize the biological functions of DEGs between the high and low-risk groups. As results, the GO function analyses revealed significant enrichment of inflammatory response-related functions, including response to molecule of bacterial origin, response to lipopolysaccharide, and positive regulation of cytokine production. Meanwhile, the DEGs were also significantly enriched in several immune-related biological processes, for instance, T cell activation and regulation of immune effector process (Fig. 7A). Besides, the KEGG pathway analyses identified that some KEGG pathways were correlative with immune-related pathways including the cytokine-cytokine receptor interaction, chemokine signaling pathway, and Toll-like receptor signaling pathway (Fig. 7B).

### Correlation of IRRS with Tumor Immune Microenvironment and Immunotherapy

The correlation of IGRS with the immune landscape of glioma microenvironment was further investigated for the immune-related functions enriched by DEGs between high and low-risk groups. Firstly, the immune system scores were compared between high and low-risk groups, and the result indicated that the low-risk group showed significantly lower immune, stromal, and ESTIMATE scores and higher tumor purity than the low-risk group (Fig. 7C–F). Then, we analyzed the different extent of immune cell infiltrations between the low- and high-risk groups for all the samples. The differences were observed in the high-risk group with lower abundance of activated NK cells, activated mast cells, naive B cells, and eosinophils, but higher abundance of CD8 + T cells, CD4 + memory activated T cells, memory B cells, plasma cells, regulatory T cells, gamma delta T cells, resting mast cells, M0-type macrophages, M1-type macrophages, and neutrophils (Fig. 7G).

**Fig. 8:**
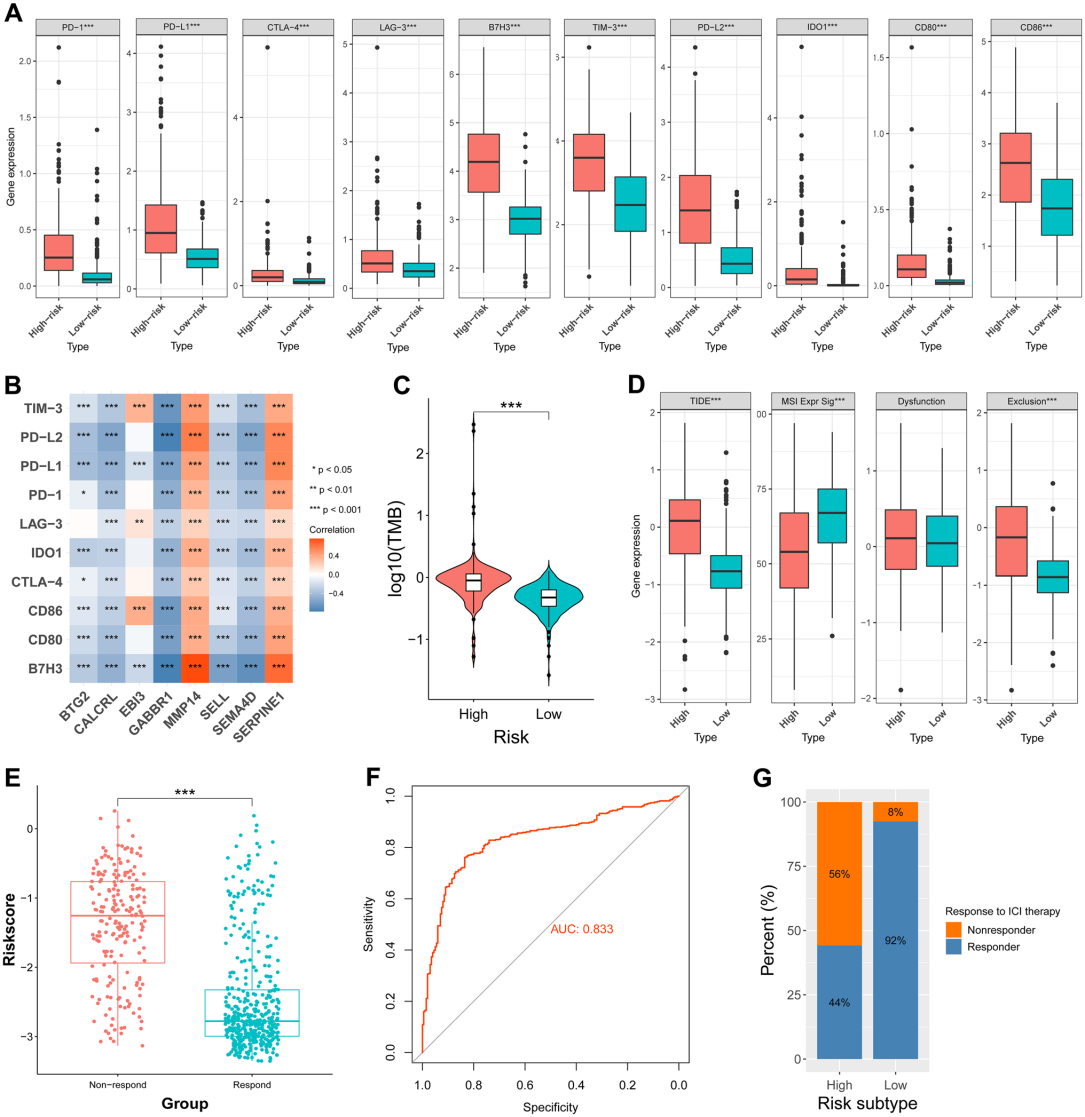
Evaluation of immune checkpoints and immunotherapy. **A** The expression levels of immune checkpoints and macrophage-associated molecules in the high and low-risk groups. ***p < 0.001. **B** Correlation analysis between the prognostic IRRS and immune checkpoints. **C** Comparative analysis of tumor mutation burden in the high- and low-risk groups. **D** The expression of TIDE, Exclusion, MSI Expe Sig, and Dysfunction between high- and low-risk groups. **E** The distributions of risk scores between the non-respond and respond groups. **F** The ROC curve of predicting immunotherapeutic benefit. **G** Comparative analysis of the response rates to ICI treatment in the high- and low-risk groups

The expressions of immune checkpoint were all upregulated in the high-risk group (Fig. 8A). We next determined whether a correlation existed between the immune checkpoint protein and prognostic inflammatory response-related genes. The correlation matrix demonstrated that the immune checkpoint protein had a significant negative correlation with the inflammatory response-related genes such as GABBR1, CALCRL, EBI3, BTG2, SEMA4D, and SELL, except for SERPINE1 and MMP14 (Fig. 8B). Moreover, a comparative analysis of TMB showed that the high-risk group had significantly higher TMB than the low-risk group (Fig. 8C).

Recently, more and more study has reported the ICIs targeting immune checkpoints such as PD-1 and PD-L1 could improve the efficiency in treatment of tumors. We found that the expression of TIDE and Exclusion was significantly higher in the high-risk group, while the expression of MSI Expe Sig was significantly lower, with the comparison to the low-risk group. However, there was no statistical difference about the expression of Dysfunction between the two groups (Fig. 8D). Then, the distributions of risk scores between the non-respond and respond groups were compared and indicated the non-respond group with significantly higher risk scores, which inferred that patient with non-response to hemotherapy had the poor prognosis (Fig. 8E). The TIDE algorithm, which was conducted to predict the immunotherapy responders through transcriptomic data, was used to explore whether prognostic genes could predict immunotherapeutic benefit in glioma. The ROC curve showed the superior predictive performance in predicting immunotherapeutic benefit (Fig. 8F). Meanwhile, the result revealed that the response rates to ICI treatment were significantly higher in low-risk patients compared with high-risk patients (*p* < 0.001, Fig. 8G). Thus, more survival benefit was likely obtained from immunotherapy for patients in the low-risk group.

### The Expression Levels of Selected Genes

**Fig. 9:**
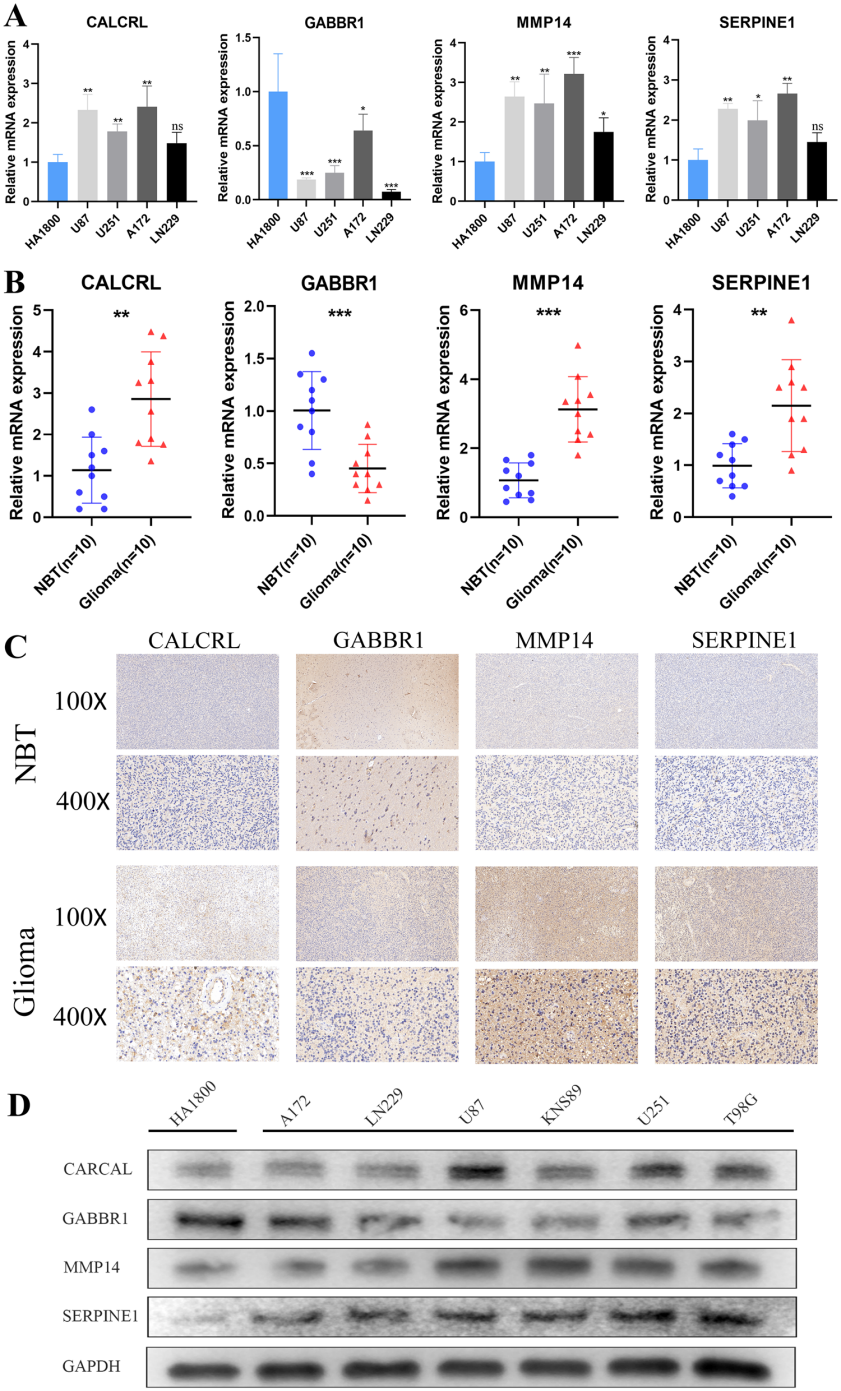
Validation of the expression levels of selected inflammatory response-related genes. **A** Scatter plots of differential transcript levels between CALCRL, GABBR1, SERPINE1, and MMP14 in glioma cell lines and normal human astrocyte cell lines. **B** Scatter plots of differential transcript levels between CALCRL, GABBR1, SERPINE1, and MMP14 in glioma and NBT. **C** The representative IHC staining images of four selective genes. **D** Western blot showed the differences of CALCRL, GABBR1, SERPINE1, and MMP14 in normal human astrocyte cell and glioma cells. *p < 0.05, **p < 0.01, ***p < 0.001, and ns no significance

We detected the expression levels of four selected inflammatory response-related genes (GABBR1, CALCRL, SERPINE1, and MMP14) in cell lines and tissue samples. As Fig. 9A, B of qRT-PCR results showed, the transcript levels of CALCRL, SERPINE1, and MMP14 were both elevated in human glioma cell lines and glioma tissues, while the transcript levels of GABBR1 exhibited an overall downward trend in human glioma cell lines and glioma tissues. The protein levels of GABBR1, CALCRL, SERPINE1, and MMP14 were qualitatively detected by IHC staining. As Fig. 9C displayed, CALCRL, SERPINE1, and MMP14 were upregulated in glioma tissues compared with NBT, whereas GABBR1 was downregulated. Western blot also showed that the protein levels of CALCRL, SERPINE1, and MMP14 in glioma cells were basically higher than human glioma cells, whereas GABBR1 has the opposite trend, which is consistent with the results qRT-PCR and IHC. The western blot results also confirmed the differences of CALCRL, GABBR1, SERPINE1, and MMP14 in normal human astrocyte cell and glioma cells. (Fig. 9D).

## Discussion

As the most common malignant brain tumor, glioma is notorious for its characteristics of low cure rate and high recurrence rate (Xu et al. 2020). Currently, the problem faced by most neurosurgeons is that the prognosis of most patients with glioma had not been improved by the existing universal treatment plan. The high heterogeneity of glioma is the main limitation, which leads to inconsistent treatment response and prognosis. Therefore, it is very important and urgent to develop personalized treatment plans for glioma patients. The rapid development of bioinformatics and sequencing technology provides a new perspective to solve this problem. Existing studies reported that hypoxia-related gene signature (Lin et al. 2020), m6A-related gene signature (Chai et al. 2019), ferroptosis-related gene signature (Zhuo et al. 2020), immune-related gene signature (Zhang et al. 2020), and energy metabolism-related gene signature (Jiang et al. 2019) are used as prognostic biomarkers to predict the prognosis of glioma. No matter the prognosis of 1-year OS, 3-year OS, or 5-year OS, they all exhibited superior predictive performance, which makes researchers confident in individually predicting the prognosis of patient by genomics. Inflammatory response, just like the above biological processes, is also an important factor in the occurrence, development, and treatment of various tumor cells (Chen et al. 2016). Lin et al. (2021) constructed a prognostic signature based on 8 inflammatory response genes to predict the prognosis of liver cancer, and the signature showed the OS of 1, 3, and 5 years with AUC is 0.685, 0.626, and 0605, respectively. Nevertheless, the inflammatory response-related gene signature as prognostic marker for glioma is still unclear.

In this study, 39 DEGs between glioma tissue and NBT were differentiated from the 200 inflammatory response-related genes. Then, 31 prognostic DEGs were identified after univariate Cox analysis; 8 of them were selected to construct prognostic IRRS. Whether in the training cohort or the validation cohorts, IRRS has been confirmed to have a strong capability in predicting survival outcomes of glioma patients. Subsequently, the prognostic IRRS was incorporated with other independent prognostic factors to construct a nomogram with superior OS prediction ability. The functional enrichment analysis revealed the potential difference in inflammatory response sensitivity between the high-risk group and the low-risk group.

Meanwhile, immune-related pathways and processes were also observed through functional enrichment analysis. Thus, we further revealed the differential immune landscape between the two risk subgroups by comparing the immune, stromal, and ESTIMATE scores, tumor purity, abundance of immune cells, and expression levels of immunoregulatory molecules. In addition, the assessment of tumor mutation burden, immune checkpoints, and immunotherapy response is also analyzed in detail based on IRRS risk stratification. To our knowledge, this is the first study to predict the prognosis of glioma with IRRS.

The IRRS constructed in our study incorporated 8 inflammatory response-related genes such as GABBR1, CALCRL, EBI3, BTG2, SEMA4D, SELL, SERPINE1, and MMP14. Except for GABBR1 and SEMA4D, other genes are upregulated in glioma tissues. Among these genes, SERPINE1 and MMP14 are associated with poor prognosis, whereas the remaining 6 genes with good prognosis. GABBR1 is a metabotropic G protein-coupled receptor that mediates the inhibitory effect of γ-aminobutyric acid; Yang et al. demonstrated that 5 miRNAs promote the proliferation and invasion of colorectal cancer cells by inhibiting GABBR1 (Longqiu et al. 2016). As a G protein-coupled seven transmembrane domain receptor, CALCRL has been identified as a potential tumor suppressor for lung adenocarcinoma by Lu et al. (2021). A β-subunit of IL-12 family member (EBI3) was confirmed to promote T- and B-cell differentiation through the gp130-STAT3 signal pathway and thereby playing a crucial role in inhibiting tumor cell proliferation and invasion (Ma et al. 2019). In the non-small-cell lung cancer models, overexpression of BTG2 prevented the lung cancer cell metastasis by inhibiting cell invasion (Chen et al. 2020). SEMA4D is an important member of the semaphorin subfamily and plays an important role in the nervous and immune systems. Chen et al. (2019) firstly reported that highly expressed SEMA4D plays an important role in osteolytic bone metastasis of lung cancer by inhibiting osteoblast differentiation, thereby providing a potential strategy for targeting osteoblasts to treat bone metastasis. However, some studies declared to promote tumor cell invasion and metastasis by tumor angiogenesis in some cancers such as prostate cancer and colon cancer (Li et al. 2018). SELL, encoding the selectin L protein (CD62L), mediates the adhesion of cells to the vascular endothelium (Dashti et al. 2018), while the role of SELL in tumorigenesis and development has not been reported yet. Conversely, SERPINE1, as a member of the superfamily encoding serine protease inhibitors, works by inhibiting proteolytic activity and promoting angiogenesis. Previous studies demonstrated that the overexpression of SERPINE1 may lead to the spread and metastasis of colon cancer and is a poor prognostic indicator for malignant tumors such as breast cancer and gastric adenocarcinoma (Li et al. 2019; Mazzoccoli et al. 2012). MMP14 is also named as membrane type 1 matrix metalloproteinase, which participates in the degradation of basement membrane and extracellular matrix and thus promotes tumor invasion and metastasis (Jin et al. 2020). It was of great significance that these genes closely correlated to the origin and metastasis of some cancers, while the detailed action mechanism remains to further investigate.

These inflammatory response related-genes are involved in the biological processes of many cancers and have predominant predictive performance as a prognostic signature. However, whether these genes affect the prognosis of glioma patients through inflammatory response remains to be clarified. The KEGG analysis revealed that DEGs between different risk subgroups were significantly enriched in pathways related to inflammatory response and cancer, which further confirmed that the inflammatory response has a close relation with tumor progression. Furthermore, the DEGs between different risk subgroups were also enriched in some immune-related biological functions such as T cell activation and regulation of immune effector process and signal pathways, which remind us that inflammatory response-related genes may be related to immunity. Thus, we subsequently analyzed the immune scores and immune cell infiltration between the two risk subgroups. Further analyses found that the high-risk group presented the positive relation with the immune scores, abundance of immunosuppressive cells (Treg, CD8 + cell) (Zhou et al. 2016), and the expression levels of immune checkpoints and macrophage-associated molecules. Conversely, tumor killer cells (activated NK cells) showed a higher abundance in the low-risk group. To some extent, these results illustrated that inflammatory response-related genes are related to the immune landscape of the glioma microenvironment. From the above results, it can be concluded that the antitumor immunity of the high-risk group is significantly weakened, and thus, we speculated that this may be an important reason for their poor prognosis. However, the potential molecular mechanism between inflammatory response-related genes and glioma immunity has not been elucidated, and further research is needed to investigate.

With the continuous improvement of gene sequencing technology in the last few decades, the knowledge of researchers for molecular structure has continued to strengthen. Cancer treatment has shifted from chemotherapy and radiotherapy targeting tumors broadly to antibody-based immunotherapies that modulate immune responses against tumors more precisely (Cao et al. 2021). ICIs, as the first generation of immunotherapy, play a pivotal role by blocking receptor and/or ligand interactions of molecules, such as PD-1/ PD-L1 pathway and CTLA-4 (Topalian et al. 2015). Some prior studies have described the therapeutic antibodies targeted therapy for immune checkpoints, which indicated robust and durable responses in patients with many cancers (Huang et al. 2020), including glioma (Yang et al. 2021). In the current study, the high-risk group had significantly higher immune checkpoints than the low-risk group, and the immune checkpoints were positively correlated with the expression of genes related to poor prognosis and negatively correlated with genes related to good prognosis. In addition, the response rate to ICIs in the low-risk group was significantly superior to the high-risk group. Therefore, our prognostic model showed superior capacity in predicting the expression level of immune checkpoints and thus guiding immunotherapy.

Certainly, there are few limitations in this study. Firstly, it is a retrospective study; thus, the results should be further validated in its clinical utility in prospective studies. Secondly, these four cohorts have various degrees of deficiencies in clinical information, especially, the Rembrandt cohort is more serious, leading to insufficient verification of some research results. In addition, the clinical practice was not able to validate for the lack of glioma samples. Thirdly, GO and KEGG enrichment analysis and subsequent analysis of immune microenvironment and immune checkpoints are only evaluated in the TCGA cohort, not verified in other cohorts. Lastly, the potential molecular mechanism between inflammatory response-related gene and immunotherapy has not yet been elucidated, and further experiments are needed to explore it.

## Conclusion

In conclusion, we performed a comprehensive analysis of the inflammatory response-related gene and established a new prognostic signature related with prognosis, glioma immune microenvironment, and immunotherapy. Accordingly, this study provided new insight for predicting prognosis, therapy selection, and follow-up of glioma patients.

### Supplementary Information

Below is the link to the electronic supplementary material.

Supplementary file1 (TIF 1681 KB)

Supplementary file2 (TIF 7673 KB)

Supplementary file3 (TIF 2702 KB)

Supplementary file4 (TIF 1736 KB)

Supplementary file5 (TIF 1885 KB)

Supplementary file6 (DOCX 45 KB)

## Data Availability

The data were analyzed by using the publicly available datasets in this study. These data can be found as follows: TCGA (https://portal.gdc.cancer.gov/), CGGA (http://www.cgga.org.cn/), and Rembrandt (http://gliovis.bioinfo.cnio.es/) websites.
