# Supplementary figures and images for: Integrative Analysis of Inflammatory Response-Related Gene for Predicting Prognosis and Immunotherapy in Glioma
Source: J Mol Neurosci. 2023 Jul 25;73(7-8):608–27. doi: 10.1007/s12031-023-02142-x (PMC10516783; doi:10.1007/s12031-023-02142-x)

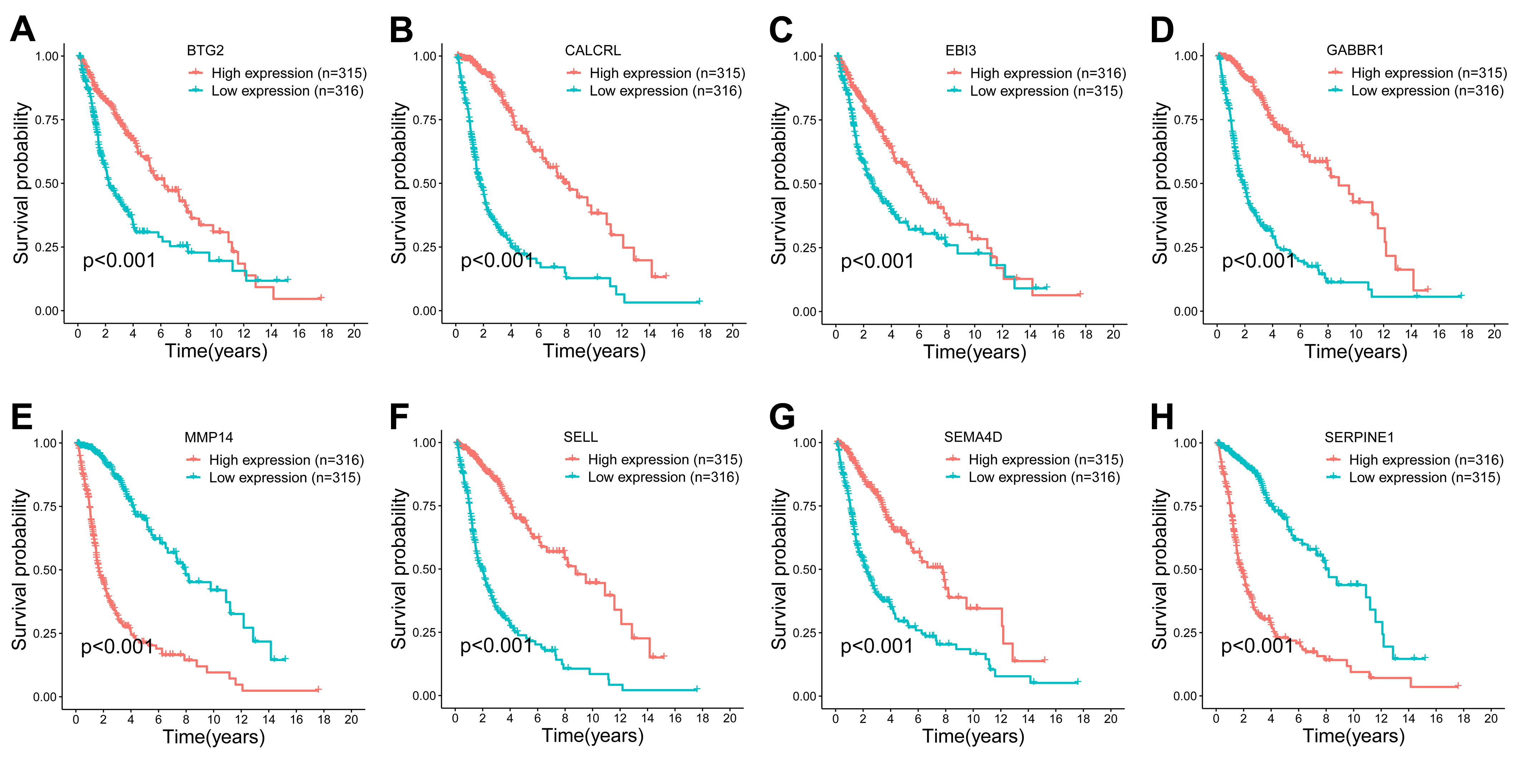

Supplement: Supplementary file 1 — Supplementary file1 (TIF 1681 KB) [file 12031_2023_2142_MOESM1_ESM.tif]

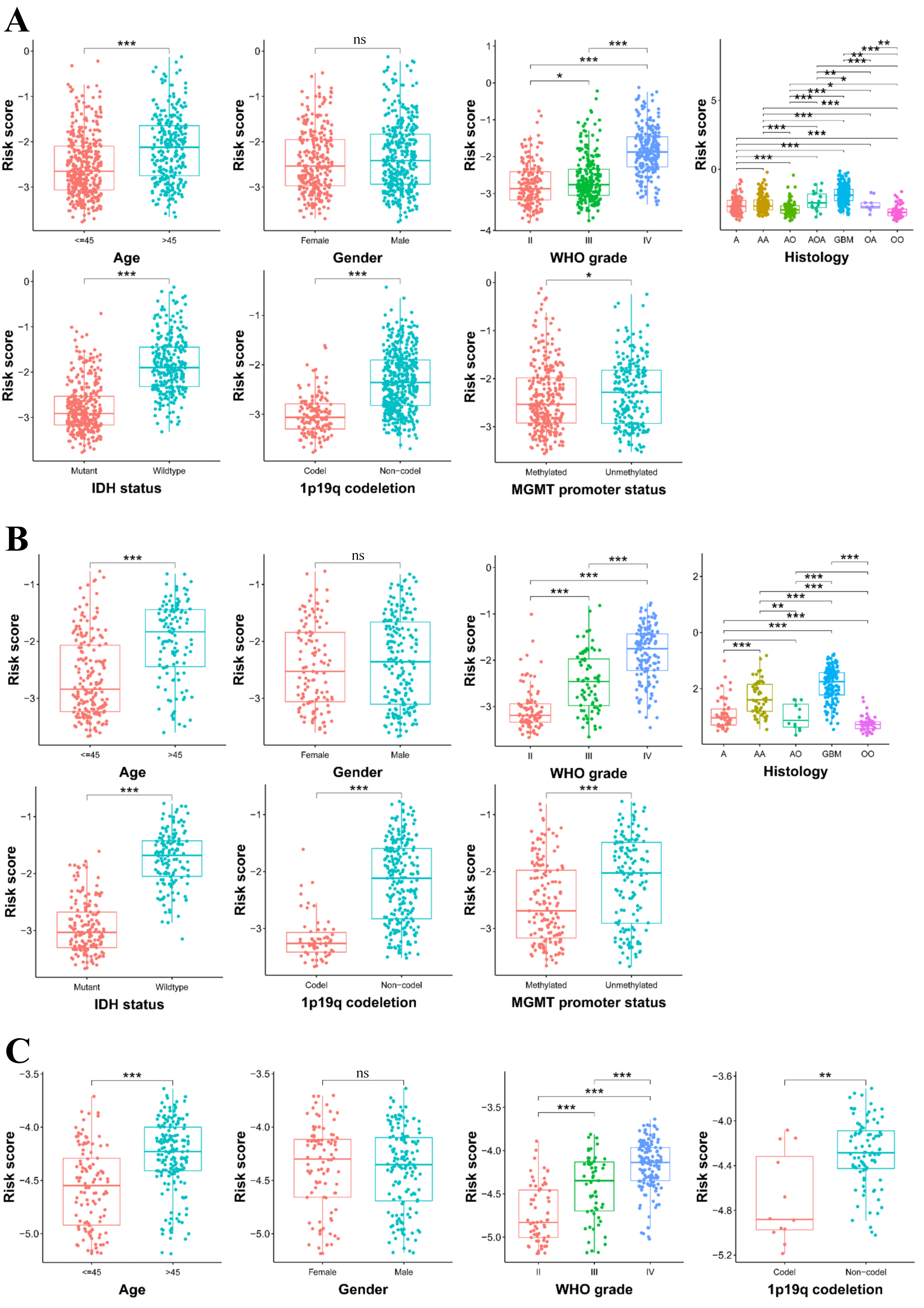

Supplement: Supplementary file 2 — Supplementary file2 (TIF 7673 KB) [file 12031_2023_2142_MOESM2_ESM.tif]

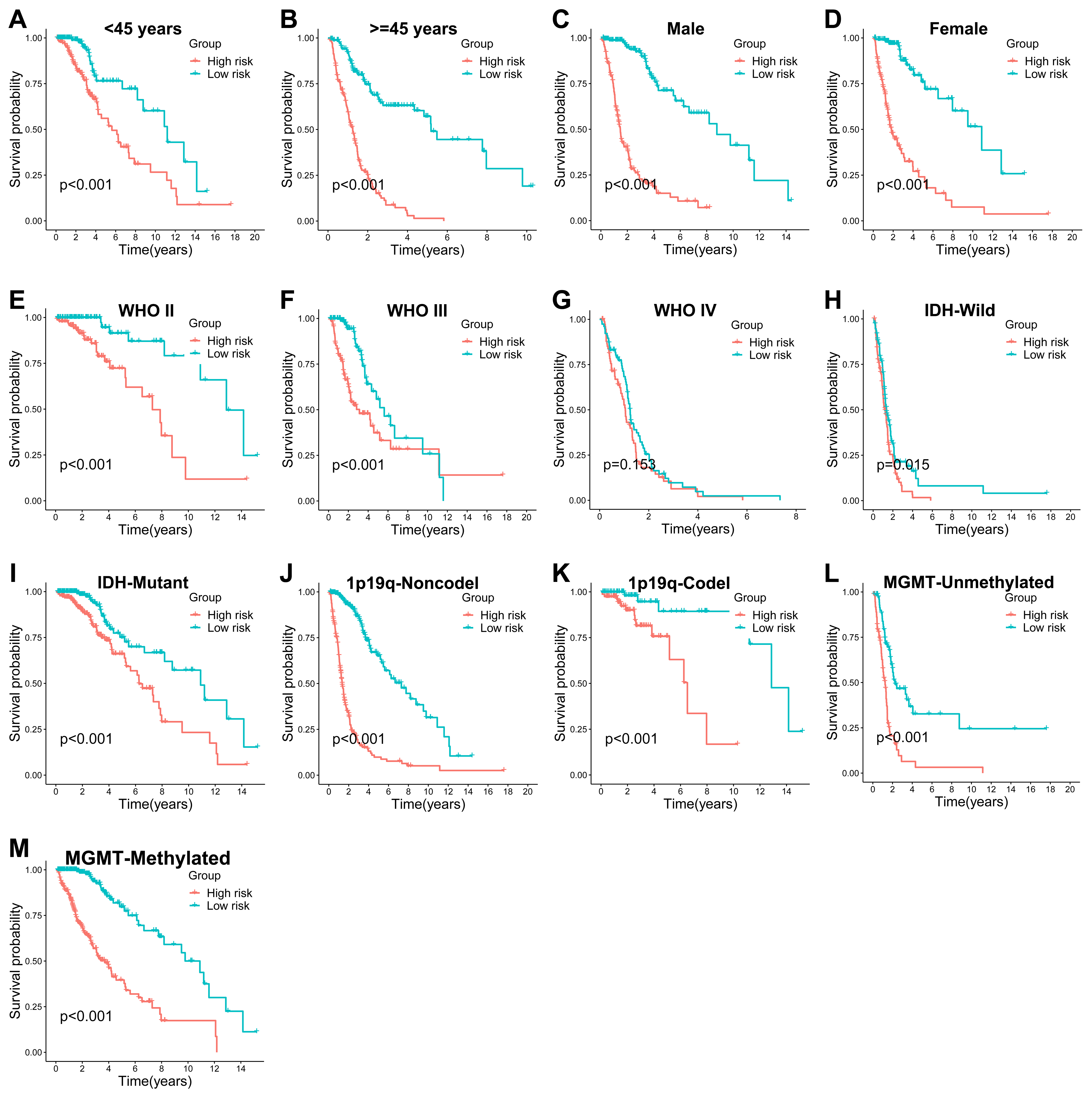

Supplement: Supplementary file 3 — Supplementary file3 (TIF 2702 KB) [file 12031_2023_2142_MOESM3_ESM.tif]

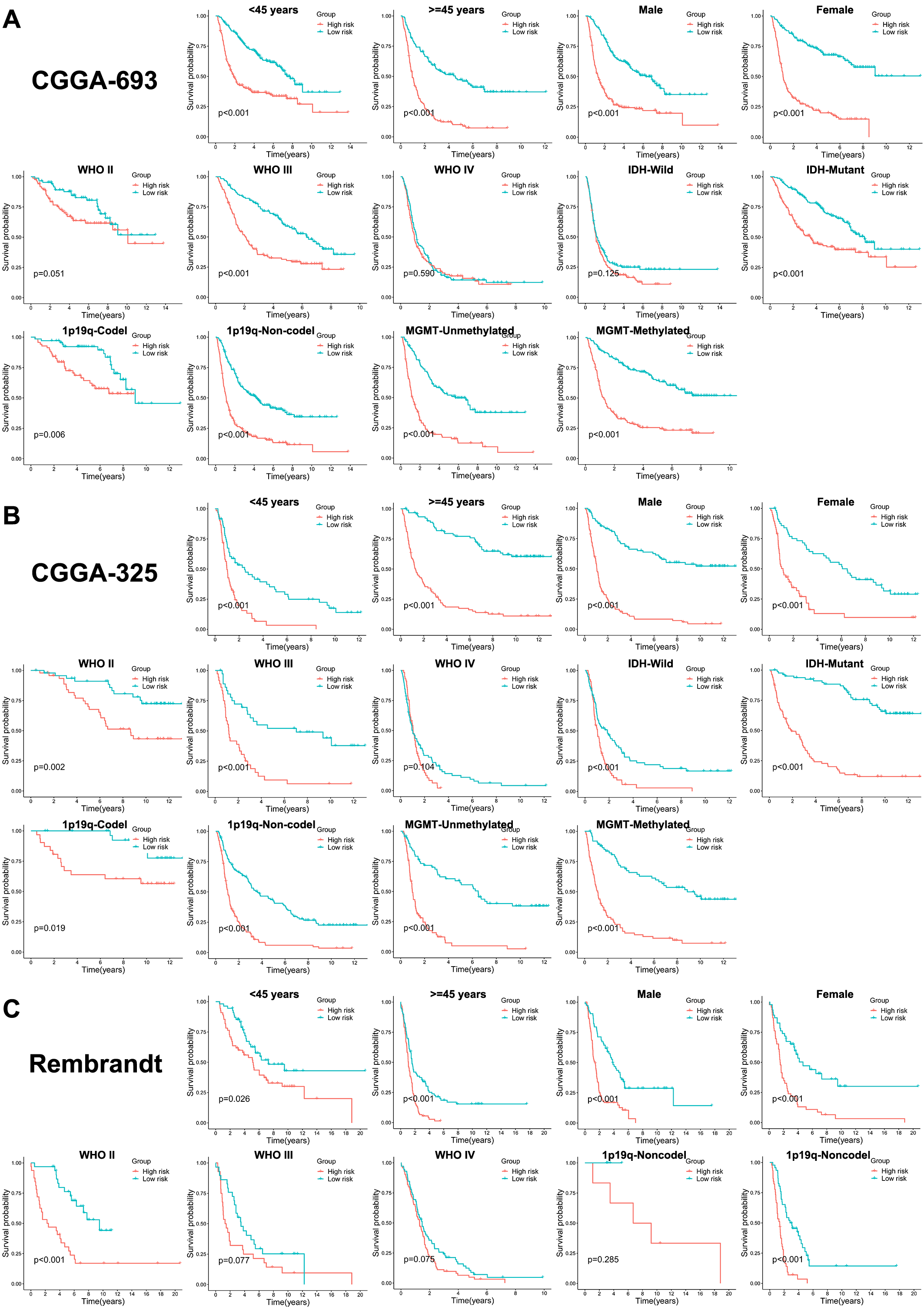

Supplement: Supplementary file 4 — Supplementary file4 (TIF 1736 KB) [file 12031_2023_2142_MOESM4_ESM.tif]

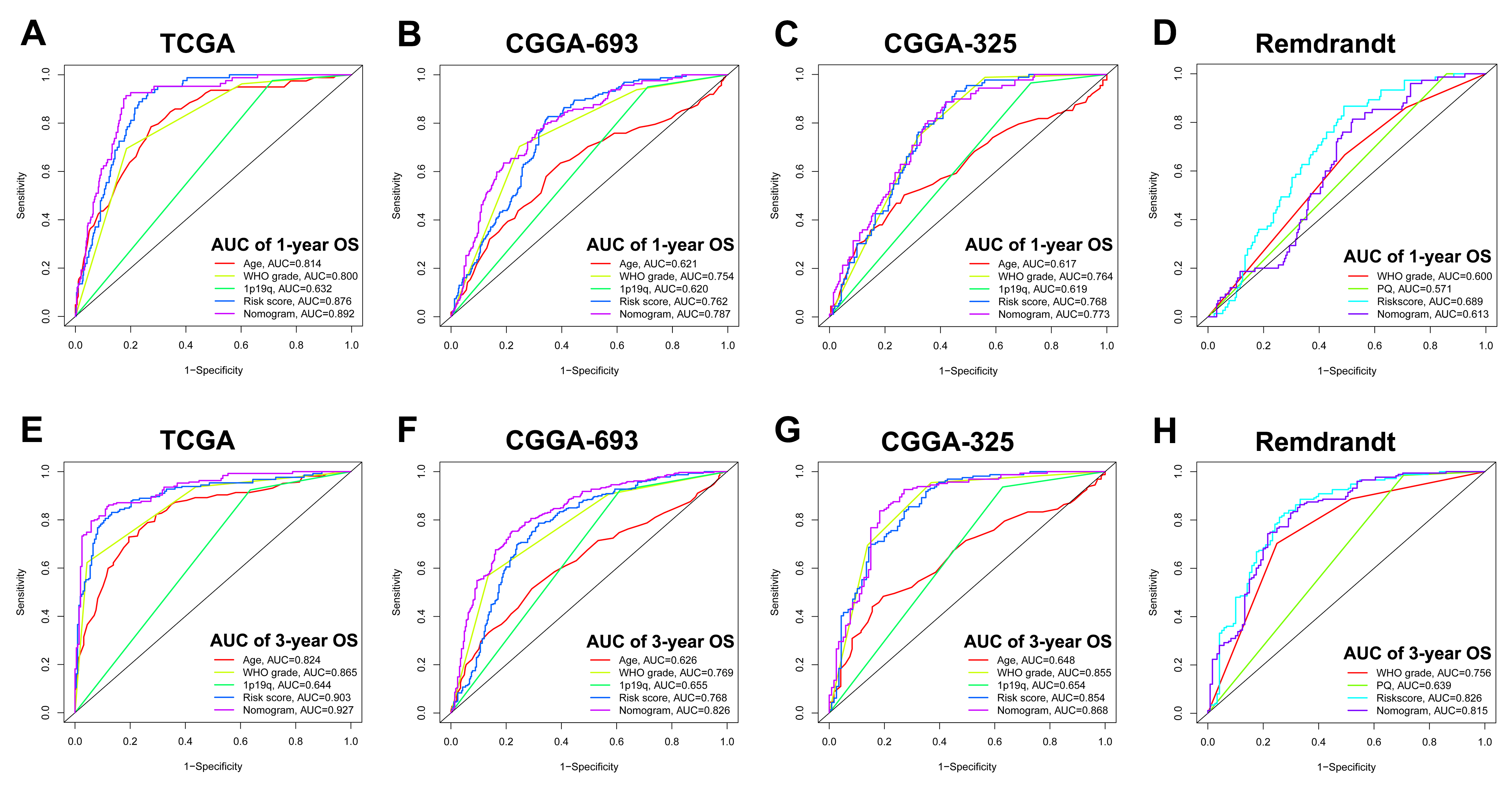

Supplement: Supplementary file 5 — Supplementary file5 (TIF 1885 KB) [file 12031_2023_2142_MOESM5_ESM.tif]
