# Supplementary material for: Integrative Analysis of Inflammatory Response-Related Gene for Predicting Prognosis and Immunotherapy in Glioma
Source: J Mol Neurosci. 2023 Jul 25;73(7-8):608–27. doi: 10.1007/s12031-023-02142-x (PMC10516783; doi:10.1007/s12031-023-02142-x)
Supplement: Supplementary file 6 — Supplementary file6 (DOCX 45 KB) [file 12031_2023_2142_MOESM6_ESM.docx]

| **Supplementary Table S1. The 200 inflammatory response-related genes** | |
| --- | --- |
| Gene ID | Gene description |
| ABCA1 | ATP binding cassette subfamily A member 1 |
| ABI1 | abl interactor 1 |
| ACVR1B | activin A receptor type 1B |
| ACVR2A | activin A receptor type 2A |
| ADM | adrenomedullin |
| ADORA2B | adenosine A2b receptor |
| ADRM1 | adhesion regulating molecule 1 |
| AHR | aryl hydrocarbon receptor |
| APLNR | apelin receptor |
| AQP9 | aquaporin 9 |
| ATP2A2 | ATPase sarcoplasmic/endoplasmic reticulum Ca2+ transporting 2 |
| ATP2B1 | ATPase plasma membrane Ca2+ transporting 1 |
| ATP2C1 | ATPase secretory pathway Ca2+ transporting 1 |
| AXL | AXL receptor tyrosine kinase |
| BDKRB1 | bradykinin receptor B1 |
| BEST1 | bestrophin 1 |
| BST2 | bone marrow stromal cell antigen 2 |
| BTG2 | BTG anti-proliferation factor 2 |
| C3AR1 | complement C3a receptor 1 |
| C5AR1 | complement C5a receptor 1 |
| CALCRL | calcitonin receptor like receptor |
| CCL17 | C-C motif chemokine ligand 17 |
| CCL2 | C-C motif chemokine ligand 2 |
| CCL20 | C-C motif chemokine ligand 20 |
| CCL22 | C-C motif chemokine ligand 22 |
| CCL24 | C-C motif chemokine ligand 24 |
| CCL5 | C-C motif chemokine ligand 5 |
| CCL7 | C-C motif chemokine ligand 7 |
| CCR7 | C-C motif chemokine receptor 7 |
| CCRL2 | C-C motif chemokine receptor like 2 |
| CD14 | CD14 molecule |
| CD40 | CD40 molecule |
| CD48 | CD48 molecule |
| CD55 | CD55 molecule (Cromer blood group) |
| CD69 | CD69 molecule |
| CD70 | CD70 molecule |
| CD82 | CD82 molecule |
| CDKN1A | cyclin dependent kinase inhibitor 1A |
| CHST2 | carbohydrate sulfotransferase 2 |
| CLEC5A | C-type lectin domain containing 5A |
| CMKLR1 | chemerin chemokine-like receptor 1 |
| CSF1 | colony stimulating factor 1 |
| CSF3 | colony stimulating factor 3 |
| CSF3R | colony stimulating factor 3 receptor |
| CX3CL1 | C-X3-C motif chemokine ligand 1 |
| CXCL10 | C-X-C motif chemokine ligand 10 |
| CXCL11 | C-X-C motif chemokine ligand 11 |
| CXCL6 | C-X-C motif chemokine ligand 6 |
| CXCL9 | C-X-C motif chemokine ligand 9 |
| CXCR6 | C-X-C motif chemokine receptor 6 |
| CYBB | cytochrome b-245 beta chain |
| DCBLD2 | discoidin, CUB and LCCL domain containing 2 |
| EBI3 | Epstein-Barr virus induced 3 |
| EDN1 | endothelin 1 |
| EIF2AK2 | eukaryotic translation initiation factor 2 alpha kinase 2 |
| EMP3 | epithelial membrane protein 3 |
| ADGRE1 | adhesion G protein-coupled receptor E1 |
| EREG | epiregulin |
| F3 | coagulation factor III, tissue factor |
| FFAR2 | free fatty acid receptor 2 |
| FPR1 | formyl peptide receptor 1 |
| FZD5 | frizzled class receptor 5 |
| GABBR1 | gamma-aminobutyric acid type B receptor subunit 1 |
| GCH1 | GTP cyclohydrolase 1 |
| GNA15 | G protein subunit alpha 15 |
| GNAI3 | G protein subunit alpha i3 |
| GP1BA | glycoprotein Ib platelet subunit alpha |
| GPC3 | glypican 3 |
| GPR132 | G protein-coupled receptor 132 |
| GPR183 | G protein-coupled receptor 183 |
| HAS2 | hyaluronan synthase 2 |
| HBEGF | heparin binding EGF like growth factor |
| HIF1A | hypoxia inducible factor 1 subunit alpha |
| HPN | hepsin |
| HRH1 | histamine receptor H1 |
| ICAM1 | intercellular adhesion molecule 1 |
| ICAM4 | intercellular adhesion molecule 4 |
| ICOSLG | inducible T cell costimulator ligand |
| IFITM1 | interferon induced transmembrane protein... |
| IFNAR1 | interferon alpha and beta receptor subun... |
| IFNGR2 | interferon gamma receptor 2 |
| IL10 | interleukin 10 |
| IL10RA | interleukin 10 receptor subunit alpha |
| IL12B | interleukin 12B |
| IL15 | interleukin 15 |
| IL15RA | interleukin 15 receptor subunit alpha |
| IL18 | interleukin 18 |
| IL18R1 | interleukin 18 receptor 1 |
| IL18RAP | interleukin 18 receptor accessory protein |
| IL1A | interleukin 1 alpha |
| IL1B | interleukin 1 beta |
| IL1R1 | interleukin 1 receptor type 1 |
| IL2RB | interleukin 2 receptor subunit beta |
| IL4R | interleukin 4 receptor |
| IL6 | interleukin 6 |
| IL7R | interleukin 7 receptor |
| CXCL8 | C-X-C motif chemokine ligand 8 |
| INHBA | inhibin subunit beta A |
| IRAK2 | interleukin 1 receptor associated kinase 2 |
| IRF1 | interferon regulatory factor 1 |
| IRF7 | interferon regulatory factor 7 |
| ITGA5 | integrin subunit alpha 5 |
| ITGB3 | integrin subunit beta 3 |
| ITGB8 | integrin subunit beta 8 |
| KCNA3 | potassium voltage-gated channel subfamily A member 3 |
| KCNJ2 | potassium inwardly rectifying channel subfamily J member 2 |
| KCNMB2 | potassium calcium-activated channel subfamily M regulatory beta subunit 2 |
| KIF1B | kinesin family member 1B |
| KLF6 | Kruppel like factor 6 |
| LAMP3 | lysosomal associated membrane protein 3 |
| LCK | LCK proto-oncogene, Src family tyrosine kinase |
| LCP2 | lymphocyte cytosolic protein 2 |
| LDLR | low density lipoprotein receptor |
| LIF | LIF interleukin 6 family cytokine |
| LPAR1 | lysophosphatidic acid receptor 1 |
| LTA | lymphotoxin alpha |
| LY6E | lymphocyte antigen 6 family member E |
| LYN | LYN proto-oncogene, Src family tyrosine kinase |
| MARCO | macrophage receptor with collagenous structure |
| MEFV | MEFV innate immuity regulator, pyrin |
| MEP1A | meprin A subunit alpha |
| MET | MET proto-oncogene, receptor tyrosine kinase |
| MMP14 | matrix metallopeptidase 14 |
| MSR1 | macrophage scavenger receptor 1 |
| MXD1 | MAX dimerization protein 1 |
| MYC | MYC proto-oncogene, bHLH transcription factor |
| NAMPT | nicotinamide phosphoribosyltransferase |
| NDP | norrin cystine knot growth factor NDP |
| NFKB1 | nuclear factor kappa B subunit 1 |
| NFKBIA | NFKB inhibitor alpha |
| NLRP3 | NLR family pyrin domain containing 3 |
| NMI | N-myc and STAT interactor |
| NMUR1 | neuromedin U receptor 1 |
| NOD2 | nucleotide binding oligomerization domain containing 2 |
| NPFFR2 | neuropeptide FF receptor 2 |
| OLR1 | oxidized low density lipoprotein receptor 1 |
| OPRK1 | opioid receptor kappa 1 |
| OSM | oncostatin M |
| OSMR | oncostatin M receptor |
| P2RX4 | purinergic receptor P2X 4 |
| P2RX7 | purinergic receptor P2X 7 |
| P2RY2 | purinergic receptor P2Y2 |
| PCDH7 | protocadherin 7 |
| PDE4B | phosphodiesterase 4B |
| PDPN | podoplanin |
| PIK3R5 | phosphoinositide-3-kinase regulatory subunit 5 |
| PLAUR | plasminogen activator, urokinase receptor |
| PROK2 | prokineticin 2 |
| PSEN1 | presenilin 1 |
| PTAFR | platelet activating factor receptor |
| PTGER2 | prostaglandin E receptor 2 |
| PTGER4 | prostaglandin E receptor 4 |
| PTGIR | prostaglandin I2 receptor |
| PTPRE | protein tyrosine phosphatase receptor type E |
| PVR | PVR cell adhesion molecule |
| RAF1 | Raf-1 proto-oncogene, serine/threonine kinase |
| RASGRP1 | RAS guanyl releasing protein 1 |
| RELA | RELA proto-oncogene, NF-kB subunit |
| RGS1 | regulator of G protein signaling 1 |
| RGS16 | regulator of G protein signaling 16 |
| RHOG | ras homolog family member G |
| RIPK2 | receptor interacting serine/threonine kinase 2 |
| RNF144B | ring finger protein 144B |
| ROS1 | ROS proto-oncogene 1, receptor tyrosine kinase |
| RTP4 | receptor transporter protein 4 |
| SCARF1 | scavenger receptor class F member 1 |
| SCN1B | sodium voltage-gated channel beta subunit 1 |
| SELE | selectin E |
| SELL | selectin L |
| SELENOS | selenoprotein S |
| SEMA4D | semaphorin 4D |
| SERPINE1 | serpin family E member 1 |
| SGMS2 | sphingomyelin synthase 2 |
| SLAMF1 | signaling lymphocytic activation molecule family member 1 |
| SLC11A2 | solute carrier family 11 member 2 |
| SLC1A2 | solute carrier family 1 member 2 |
| SLC28A2 | solute carrier family 28 member 2 |
| SLC31A1 | solute carrier family 31 member 1 |
| SLC31A2 | solute carrier family 31 member 2 |
| SLC4A4 | solute carrier family 4 member 4 |
| SLC7A1 | solute carrier family 7 member 1 |
| SLC7A2 | solute carrier family 7 member 2 |
| SPHK1 | sphingosine kinase 1 |
| SRI | sorcin |
| STAB1 | stabilin 1 |
| TACR1 | tachykinin receptor 1 |
| TACR3 | tachykinin receptor 3 |
| TAPBP | TAP binding protein |
| TIMP1 | TIMP metallopeptidase inhibitor 1 |
| TLR1 | toll like receptor 1 |
| TLR2 | toll like receptor 2 |
| TLR3 | toll like receptor 3 |
| TNFAIP6 | TNF alpha induced protein 6 |
| TNFRSF1B | TNF receptor superfamily member 1B |
| TNFRSF9 | TNF receptor superfamily member 9 |
| TNFSF10 | TNF superfamily member 10 |
| TNFSF15 | TNF superfamily member 15 |
| TNFSF9 | TNF superfamily member 9 |
| TPBG | trophoblast glycoprotein |
| VIP | vasoactive intestinal peptide |

**Supplementary Table S2. Univariate COX regression analyses of DEGs**

| **Genes** | **Hazard ratio** | ***p-*value** |
| --- | --- | --- |
| ABCA1 | 0.644(0.554−0.749) | <0.001 |
| APLNR | 1.042(0.963−1.127) | 0.307 |
| ATP2B1 | 0.790(0.686−0.910) | 0.001 |
| ATP2C1 | 0.250(0.197−0.316) | <0.001 |
| BEST1 | 0.402(0.335−0.482) | <0.001 |
| BTG2 | 0.549(0.477−0.633) | <0.001 |
| C3AR1 | 1.198(1.045−1.374) | 0.010 |
| CALCRL | 0.582(0.534−0.633) | <0.001 |
| CD14 | 1.305(1.191−1.431) | <0.001 |
| CD55 | 0.440(0.330−0.587) | <0.001 |
| CMKLR1 | 0.370(0.296−0.461) | <0.001 |
| CSF1 | 0.714(0.601−0.847) | <0.001 |
| CSF3R | 1.036(0.803−1.338) | 0.783 |
| CYBB | 1.202(1.057−1.368) | 0.005 |
| EBI3 | 0.531(0.433−0.650) | <0.001 |
| FPR1 | 1.488(1.338−1.655) | <0.001 |
| GABBR1 | 0.574(0.535−0.615) | <0.001 |
| HAS2 | 1.226(1.051−1.430) | 0.009 |
| HIF1A | 0.690(0.583−0.816) | <0.001 |
| HPN | 0.358(0.251−0.511) | <0.001 |
| ICOSLG | 0.599(0.289−1.244) | 0.169 |
| IFITM1 | 1.023(0.917−1.142) | 0.679 |
| LPAR1 | 1.120(1.002−1.250) | 0.045 |
| LY6E | 1.077(0.948−1.224) | 0.253 |
| MMP14 | 1.904(1.729−2.097) | <0.001 |
| MYC | 0.624(0.559−0.697) | <0.001 |
| OLR1 | 1.223(1.086−1.377) | <0.001 |
| P2RX4 | 0.344(0.232−0.509) | <0.001 |
| PTAFR | 0.888(0.740−1.066) | 0.202 |
| SCN1B | 0.477(0.401−0.567) | <0.001 |
| SELL | 0.661(0.610−0.716) | <0.001 |
| SEMA4D | 0.366(0.308−0.435) | <0.001 |
| SERPINE1 | 1.584(1.482−1.694) | <0.001 |
| SGMS2 | 0.549(0.328−0.919) | 0.023 |
| SLC31A2 | 0.469(0.357−0.615) | <0.001 |
| SLC4A4 | 0.651(0.596−0.710) | <0.001 |
| TPBG V | 0.883(0.684−1.138) | 0.336 |
| VIP | 0.505(0.409−0.624) | <0.001 |

**Supplementary Table S3. The comparison of clinicopathological characteristics between the high-risk and low-risk groups in the TCGA cohort**

| **Clinicopathological characteristics** | **High-risk group**  **(n = 315)** | **Low-risk group**  **(n = 316)** | ***p-*value** |
| --- | --- | --- | --- |
| **Age** (median [IQR]) | 56.0 [43.0,64.0] | 39.0 [32.0,48.0] | <0.001 |
| **Gender** |  |  | 0.078 |
| Male | 181 (61.36%) | 152 (54.09%) |  |
| Female | 114 (38.64%) | 129 (49.91%) |  |
| **Histology** |  |  | <0.001 |
| Astrocytoma | 79 (26.78%) | 75 (26.69%) |  |
| Oligoastrocytoma | 34 (11.53%) | 76 (27.05%) |  |
| Oligodendroglioma | 31 (10.51%) | 128 (45.56%) |  |
| Glioblastoma | 151 (51.19%) | 2 (0.71%) |  |
| **WHO grade** |  |  | <0.001 |
| II | 40 (13.56%) | 161 (57.30%) |  |
| III | 104 (35.24%) | 118 (41.99%) |  |
| IV | 151 (51.19%) | 2 (0.71%) |  |
| **IDH status** |  |  | <0.001 |
| Mutant | 93 (31.10%) | 308 (98.40%) |  |
| Wild type | 216 (69.90%) | 5 (1.60%) |  |
| **1p/19q codeletion** |  |  | <0.001 |
| Codel | 19 (6.15%) | 138 (43.67%) |  |
| Non-codel | 290 (93.85.7%) | 178 (56.33%) |  |
| **MGMT promoter status** |  |  | <0.001 |
| Methylated | 157(55.28%) | 292 (92.70%) |  |
| Unmethylated | 127 (44.72%) | 23 (7.30%) |  |

**Supplementary Table S4. The comparison of clinicopathological characteristics between the high-risk and low-risk groups in the CGGA-693 cohort**

| **Clinicopathological characteristics** | **High-risk group**  **(n = 328)** | **Low-risk group**  **(n = 328)** | ***p-*value** |
| --- | --- | --- | --- |
| **Age** (Mean ± SD) | 46.0 ± 13.6 | 40.8 ± 10.5 | <0.001 |
| **Gender** |  |  | 0.156 |
| Male | 196 (59.76%) | 178 (54.27%) |  |
| Female | 132 (40.24%) | 150 (45.73%) |  |
| **WHO grade** |  |  | <0.001 |
| II | 51 (15.55%) | 121 (36.89%) |  |
| III | 78 (23.78%) | 170 (51.83%) |  |
| IV | 199 (60.67%) | 37 (11.28%) |  |
| **IDH status** |  |  | <0.001 |
| Mutant | 76 (24.28%) | 256 (86.78%) |  |
| Wild type | 237 (75.72%) | 39 (13.22%) |  |
| **1p/19q codeletion** |  |  | <0.001 |
| Codel | 12 (4.53%) | 125 (38.46%) |  |
| Non-codel | 253 (95.48%) | 200 (61.53%) |  |
| **MGMT promoter status** |  |  | 0.085 |
| Methylated | 142 (54.62%) | 162 (62.07%) |  |
| Unmethylated | 118 (45.39%) | 99 (37.93%) |  |

**Supplementary Table S5. The comparison of clinicopathological characteristics between the high-risk and low-risk groups in the CGGA-325 cohort**

| **Clinicopathological characteristics** | **High-risk group**  **(n = 154)** | **Low-risk group**  **(n = 155)** | ***p-*value** |
| --- | --- | --- | --- |
| **Age** (Mean ± SD) | 47.16 ± 12.98 | 39.40 ± 9.24 | <0.001 |
| **Gender** |  |  | 0.310 |
| Male | 101 (65.58%) | 93 (60.0%) |  |
| Female | 53 (34.42%) | 62 (40.0%) |  |
| **WHO grade** |  |  | <0.001 |
| II | 10 (6.67%) | 81 (56.13%) |  |
| III | 32 (21.33%) | 47 (26.45%) |  |
| IV | 108 (72.0%) | 21 (17.42%) |  |
| **IDH status** |  |  | <0.001 |
| Mutant | 27 (17.53%) | 138 (89.61%) |  |
| Wild type | 127 (82.47%) | 16 (10.39%) |  |
| **1p/19q codeletion** |  |  | <0.001 |
| Codel | 4 (2.69%) | 58 (38.16%) |  |
| Non-codel | 145 (97.32%) | 94 (61.84%) |  |
| **MGMT promoter status** |  |  | <0.001 |
| Methylated | 60 (40.54%) | 91 (63.64%) |  |
| Unmethylated | 88 (59.46%) | 52 (36.36%) |  |

**Supplementary Table S6. The comparison of clinicopathological characteristics between the high-risk and low-risk groups in the Rembrandt cohort**

| **Clinicopathological characteristics** | **High-risk group**  **(n = 150)** | **Low-risk group**  **(n = 150)** | ***p-*value** |
| --- | --- | --- | --- |
| **Age** |  |  | <0.001 |
| <=45 | 30 (20.41%) | 80 (55.56%) |  |
| >45 | 117 (79.59%) | 64 (44.44%) |  |
| **Gender** |  |  | 0.426 |
| Male | 70 (58.82%) | 76 (63.86%) |  |
| Female | 49 (41.18%) | 43 (36.13%) |  |
| **WHO grade** |  |  | <0.001 |
| II | 10 (6.92%) | 56 (45.39%) |  |
| III | 28 (19.45%) | 29 (23.58%) |  |
| IV | 106 (73.61%) | 38 (30.89%) |  |
| **1p/19q codeletion** |  |  | 0.025 |
| Codel | 3 (5.26%) | 9 (19.57%) |  |
| Non-codel | 54 (94.74%) | 37 (80.43%) |  |
